# Host GRXC6 restricts *Tomato yellow leaf curl virus* infection by inhibiting the nuclear export of the V2 protein

**DOI:** 10.1371/journal.ppat.1009844

**Published:** 2021-08-16

**Authors:** Wenhao Zhao, Yijun Zhou, Xueping Zhou, Xiaofeng Wang, Yinghua Ji

**Affiliations:** 1 Key Lab of Food Quality and Safety of Jiangsu Province—State Key Laboratory Breeding Base, Institute of Plant Protection, Jiangsu Academy of Agricultural Sciences, Nanjing, China; 2 School of Plant and Environmental Sciences, Virginia Tech, Blacksburg, Virginia, United States of America; 3 State Key Laboratory for Biology of Plant Disease and Insect Pests, Institute of Plant Protection, Chinese Academy of Agricultural Sciences, Beijing, China; 4 State Key Laboratory of Rice Biology, Institute of Biotechnology, Zhejiang University, Hangzhou, China; The Ohio State University, UNITED STATES

## Abstract

Geminiviruses cause serious symptoms and devastating losses in crop plants. With a circular, single-stranded DNA genome, geminiviruses multiply their genomic DNA in the nucleus, requiring the nuclear shuttling of viral proteins and viral genomic DNAs. Many host factors, acting as proviral or antiviral factors, play key roles in geminivirus infections. Here, we report the roles of a tomato glutaredoxin (GRX), SlGRXC6, in the infection of *Tomato yellow leaf curl virus* (TYLCV), a single-component geminivirus. The V2 protein of TYLCV specifically and preferentially interacts with SlGRXC6 among the 55-member tomato GRX family that are broadly involved in oxidative stress responses, plant development, and pathogen responses. We show that overexpressed SlGRXC6 increases the nuclear accumulation of V2 by inhibiting its nuclear export and, in turn, inhibits trafficking of the V1 protein and viral genomic DNA. Conversely, the silenced expression of SlGRXC6 leads to an enhanced susceptibility to TYLCV. SlGRXC6 is also involved in symptom development as we observed a positive correlation where overexpression of SlGRXC6 promotes while knockdown of SlGRXC6 expression inhibits plant growth. We further showed that SlGRXC6 works with SlNTRC80, a tomato NADPH-dependent thioredoxin reductase, to regulate plant growth. V2 didn’t interact with SlNTRC80 but competed with SlNTR80 for binding to SlGRXC6, suggesting that the V2-disrupted SlGRXC6-SlNTRC80 interaction is partially responsible for the virus-caused symptoms. These results suggest that SlGRXC6 functions as a host restriction factor that inhibits the nuclear trafficking of viral components and point out a new way to control TYLCV infection by targeting the V2-SlGRXC6 interaction.

## Author summary

Geminiviruses infect numerous crops, induce a wide range of symptoms, and cause tremendous crop losses annually. *Tomato yellow leaf curl virus* (TYLCV), a single-component geminivirus, is a causative agent leading to one of the most devastating tomato diseases in the world. As a single-stranded DNA virus, genomic replication occurs in the nucleus and therefore, the nuclear shuttling is a critical step of viral infection. The V2 protein of TYLCV is involved in symptom development and viral trafficking, among other steps, and hijacks host proteins for executing its functions. Nevertheless, host factors involved in the V2-mediated functions are not well addressed. We show that tomato GRXC6 (SlGRXC6) functions as a restriction factor of TYLCV infection by interacting with and preventing V2 from moving out of the nucleus, leading to the inhibited V2-mediated nuclear export of V1 and the V1-viral DNA complex. SlGRXC6 also contributes to symptom development via its interaction with SINTRC80. V2 sequesters SlGRXC6 from forming the SlGRXC6-SlNTRC80 complex and regulates plant growth. Our work, therefore, identified a new host partner of V2 and revealed the mechanisms whereby V2 functions as a pathogenicity determinant and can be targeted for virus control.

## Introduction

Geminiviruses are a group of plant viruses with a circular, single-stranded DNA genome that is encapsulated in twinned icosahedral particles [1–3]. Viral infection causes disease symptoms that include chlorosis and/or necrosis, leaf curling, and altered plant stature and morphology, leading to extensive agricultural losses worldwide [2,4,5].

Geminiviruses encode 4 to 7 proteins, with several contributing to pathogenicity. These proteins differ between monopartite and bipartite geminiviruses, as well as between viruses within the individual groups. For example, the C4 protein of *Tomato leaf curl virus* (ToLCV) and βC1 from other monopartite geminiviruses are identified as a pathogenicity determinant [6–11]. V2 protein is also involved in symptom development in host plants because curly leaves and yellowing veins are observed on systemic leaves in plants overexpressing V2 from *African cassava mosaic virus* (ACMV), *East African cassava mosaic Cameroon virus* (EACMCV), *Cotton leaf curl Multan virus* (CLCuMV), *Beet curly top virus* (BCTV) and ToLCV [6,12–16], indicating that the V2 protein of geminiviruses is an important symptom determinant.

*Tomato yellow leaf curl virus* (TYLCV) is a typical monopartite begomovirus in the family *Geminiviridae* that contains a single genome component with six open reading frames (ORFs). Multiple studies have shown that V2 is a suppressor of gene silencing at both the post-transcriptional stage (PTGS) [17,18] and the transcriptional level (TGS) [19,20]. V2 is also involved in the regulation of host defence responses [21] and viral movement [2,22–26], playing important roles in viral spread and systemic infection [27,28]. Moreover, it was found that V2, when expressed from a PVX vector, caused severe leaf curling, stunting, vein yellowing and necrotic lesions on systemic leaves [13,19,29], suggesting that V2 may serve as an important symptom determinant in TYLCV pathogenicity. Nevertheless, it is unclear how and what host factors are involved in the V2-mediated functions during TYLCV infection.

Glutaredoxins (GRXs) are a group of low-molecular-weight thiol oxidoreductasea and are implicated in response to oxidative stress [30]. Based on the sequences of the active site, GRXs are classified into three distinct subgroups: the CPYC type, which contains C[P/G/S][Y/F][C/S] motifs; the CGFS type, which has a strictly conserved CGFS active site; and the CC type, which contains the CC[M/L][C/S/G/A/I] active site [31]. While the CPYC- and CGFS-type GRXs are widely found in all eukaryotic and prokaryotic species, the CC-type GRXs are restricted to land plants [32]. In plants, GRXs are involved in plant development, signal transduction, and other biological processes [30,33,34]. It has recently been reported that GRXs play an important role in response to pathogens [34], but the mechanism is not well known.

Tomato (*Solanum lycopersicum*) glutaredoxin-C6 (SlGRXC6) belongs to the CC-type GRXs, which is the largest type in plants [31,35]. Early reports show that CC-type GRXs are involved in plant development [36] and pathogen responses through jasmonic acid (JA) or salicylic acid (SA) signalling pathways [34,37]. Among the five well-studied CC-type GRXs in *Arabidopsis*, three are related to host defence response: ROXY19 (GRX480), ROXY4 and GRXS13 [38–40]. The other two (ROXY1 and ROXY2) are involved in flower development and regulating floral organ primordium formation [36,41–43]. The two CC-type GRXs in rice (OsROXY1 and OsROXY2) are also involved in defence responses to pathogens [44]. These collectively suggest that the CC-type GRXs play an important role in plant growth and host defence responses to pathogens.

It is well-known that there are cross-talks between the thioredoxin (TRX) and the glutathione systems [45]. TRXs and GRXs are critical for plant development and cell division. They also act as key signalling molecules in response to abiotic and biotic stresses [46–49]. The interrelation between GRX and NADPH-dependent thioredoxin reductase (NTR) in the regulation of plant growth has previously been investigated [50,51]. In *Arabidopsis*, the overexpression of NTR enhanced leaf growth and the overall height of plants, which was about 40% higher than that of wild-type (WT) plants [52]. Conversely, a lack of NTR results in stunted growth and decreased fertility [51,53,54]. On the other hand, the glutathione pathway plays a major role in compensating for NTR inactivation in the mutant transgenic plants [55]. In addition, plants lacking NTR show enhanced disease susceptibility to fungal and bacterial pathogens [56,57]. These results suggest that GRXs may work together with NTR in regulating plant growth and response to pathogen attacks; however, there is no direct evidence supporting this claim.

In this study, we demonstrate that SlGRXC6 is a host restriction factor of TYLCV infection. We identify and characterize a specific interaction between V2 and SlGRXC6, and show that overexpressed SlGRXC6 inhibits the nuclear export of V2 and, in turn, inhibits viral systemic infection in tomato plants. Conversely, knocking down the expression of *SlGRXC6* promotes viral infection. We additionally demonstrate that expression levels of SlGRXC6 have a positive correlation with plant growth and that the SlGRXC6-mediated plant growth is related to SlNTRC80, which interacts with SlGRXC6. Using a competitive pulldown assay, we demonstrate that V2 competes with SlNTRC80 for a direct binding to SlGRXC6, suggesting that the disrupted SlGRXC6-SlNTRC80 interaction is likely accountable for the affected host growth during viral infection. These results show that SlGRXC6 is a host restriction factor that prohibits TYLCV infection and that regulating the V2-SlGRXC6 interaction could provide a new way to control TYLCV.

## Results

### The V2 protein Interacts with SlGRXC6

**Fig 1:**
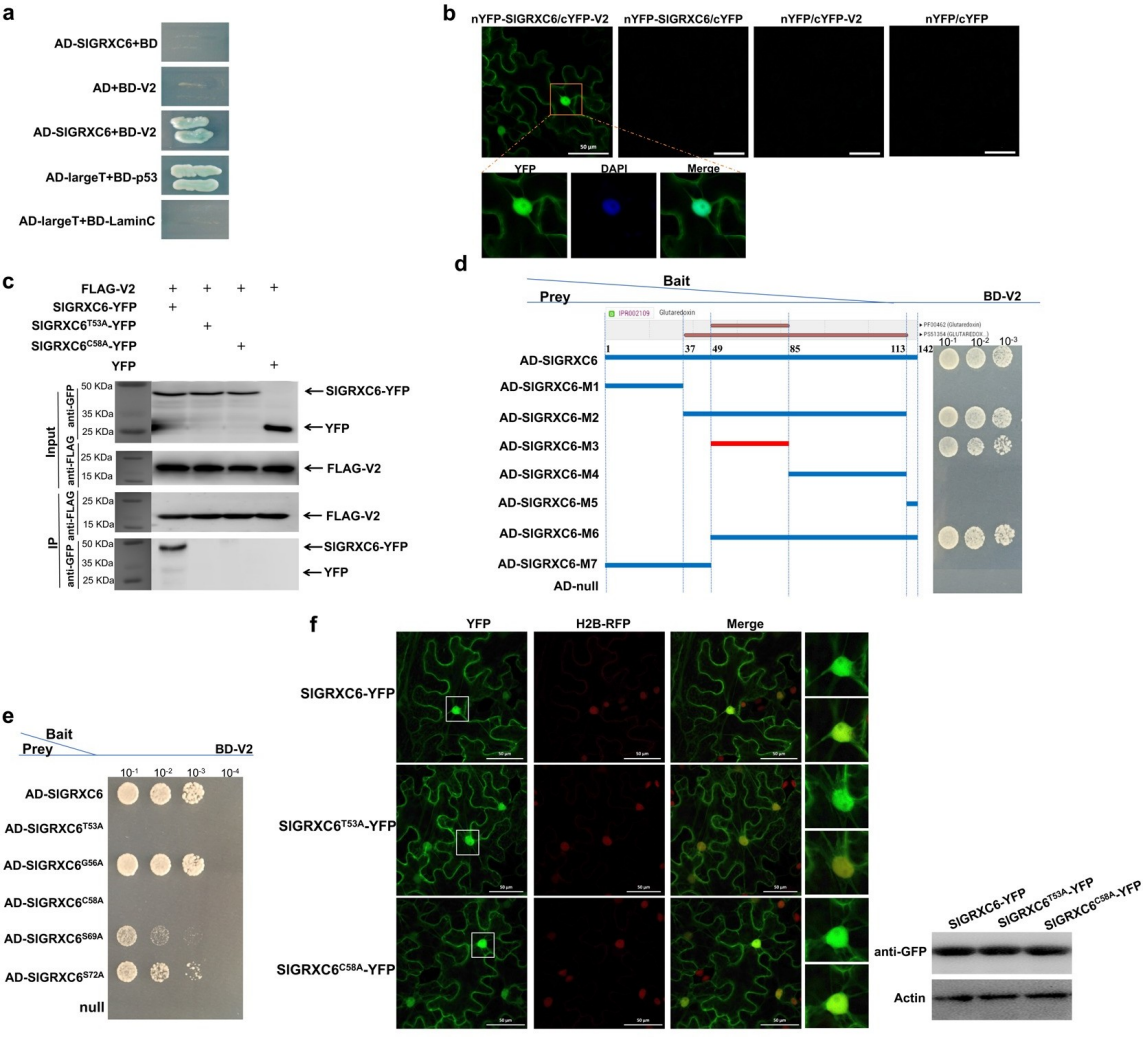
Identification of the interaction between V2 and SlGRXC6 proteins. (a) Y2H confirmed the interaction between V2 and SlGRXC6. SlGRXC6 was fused with a GAL4 activation domain (AD-SlGRXC6) and V2 was fused to a GAL4-binding domain (BD-V2), respectively. Yeast cells expressing the indicated protein pairs were plated onto the selection medium (SD/-His/-Leu/-Trp/-Ade) with X-α-Gal to screen for positive interactions. Yeast cells coexpressing pGADT7-largeT (AD-largeT) and pGBKT7-p53 (BD-p53) or AD-largeT and pGBKT7-LaminC (BD-LaminC) served as a positive and negative control, respectively. (b) The V2-SlGRXC6 interaction was confirmed by using a BiFC assay in *N*. *benthamiana* cells. The V2-SlGRXC6 interaction led to a reconstituted fluorescence signal. DAPI stains DNA in the nucleus. Bars: 50 μm. Experiments were repeated three times and 30 cells were observed in each repeat. (c) A co-IP assay to test the interaction between V2 and SlGRXC6, SlGRXC6^T53A^ or SlGRXC6^C58A^. *N*. *benthamiana* leaves were co-infiltrated with FLAG-V2 and SlGRXC6-YFP (Lane 1), FLAG-V2 and SlGRXC6^T53A^-YFP (Lane 2), FLAG-V2 and SlGRXC6^C58A^-YFP (Lane 3), or FLAG-V2 and YFP (Lane 4). Cell lysates were incubated with FLAG-Trap beads and proteins pulled down with beads were tested using the indicated antibodies. Samples before (Input) and after (IP) immunoprecipitation were analyzed by using anti-GFP or -FLAG antibody. (d) Schematic representation of the truncated mutants of SlGRXC6 and their interactions with V2 as analyzed using Y2H. (e) Interactions between the V2 and the SlGRXC6 mutants SlGRXC6^T53A^, SlGRXC6^G56A^, SlGRXC6^C58A^, SlGRXC6^S69A^, SlGRXC6^S72A^ using Y2H. (f) Subcellular localization of SlGRXC6-YFP, SlGRXC6^T53A^-YFP or SlGRXC6^C58A^-YFP in H2B transgenic *N*. *benthamiana*. The H2B-RFP signal represents the nucleus. Bars: 50 μm. Experiments were repeated three times with similar results.

It has been reported that the expression of TYLCV V2 protein induced severe symptom-like phenotype in plants [13,19,29]. We observed a similar phenotype in tomato transgenic plants expressing V2 of TYLCV (S1 Fig), indicating that V2 plays an important role in viral pathogenicity. To identify host target(s) involved in the V2-mediated pathogenesis in host plants, we performed a yeast two-hybrid (Y2H) screen of a tomato cDNA library using V2 as the bait. Two cDNAs encoding full-length SlGRXC6 (GenBank accession no. XM004251147) were identified, suggesting that SlGRXC6 could be an interacting partner of V2. To further confirm the SlGRXC6-V2 interaction, the full-length coding sequence of SlGRXC6 was amplified using tomato leaf tissue as a source and its interaction with V2 was confirmed in Y2H (Fig 1A).

A bimolecular fluorescence complementation (BiFC) assay was performed to verify the interaction between V2 and SlGRXC6 in plant cells. SlGRXC6 and V2 were fused to the N-terminal and C-terminal fragments of yellow fluorescent protein (YFP), respectively. The corresponding constructs were co-delivered into *N*. *benthamiana* leaves by agroinfiltration and fluorescence was observed using a confocal microscope at 48 hours post-agroinfiltration (hpai). A positive interaction between nYFP-SlGRXC6 and cYFP-V2 was observed in both the cytoplasm and the nucleus as indicated by the presence of the reconstituted fluorescence in all cells (n = 30) (Fig 1B). No fluorescence signal was generated when nYFP-SlGRXC6 and cYFP or nYFP and cYFP-V2 were coexpressed (Fig 1B), reinforcing a specific interaction between V2 and SlGRXC6 in plant cells.

To provide more evidence of the interaction between V2 and SlGRXC6, a co-immunoprecipitation (co-IP) assay was performed with coexpressed FLAG-tagged V2 (FLAG-V2) and YFP or YFP-tagged SlGRXC6 (SlGRXC6-YFP) in *N*. *benthamiana* leaves. Total protein extracts were incubated with FLAG-Trap beads and the resulting precipitates were analysed by western blot assays using anti-FLAG or -YFP antibody. We found that SlGRXC6-YFP, but not YFP, co-precipitated with FLAG-V2 (Fig 1C), even though both YFP and SlGRXC6-YFP were well-expressed (Fig 1C, top panel). These results indicate a specific association between V2 and SlGRXC6 in plant cells.

In tomato, there are 55 GRXs that are divided into four groups; the CC-type is the largest type with 35 members [35]. Amino acid sequence analysis revealed that SlGRXC6 is a close homolog of AtGRXC10 and AtGRXC6 and belongs to the CC-type GRXs with a conserved CCMC motif (S2A Fig). To test whether SlGRXC6 was the only one among the tomato GRXs that specifically interacted with V2, we selected multiple members to test their interactions with V2 (S2B Fig). Three members were from the CC-type GRXs: SlGRX25, SlGRX38, and SlGRX39. SlGRX38 and SlGRX39 have the same CCMC active motif as SlGRXC6, but SlGRX25 has a CCIS active motif. We also included two GRXs from the CGFS-type (SlGRX9 and SlGRX36), two from the CYPC-type (SlGRX27 and SlGRX43), and two from the GRL-type (SlGRXL1 and SlGRXL3) [35]. As shown in S2B Fig, all yeast cells expressing V2 and one of different tomato GRXs grew well on medium without selection, but most did not grow at all on the selection medium. Yeast cells expressing V2 and SlGRX43 or SlGRX39 grew much slower than those with V2 and SlGRXC6, showing that V2 preferentially interacts with SlGRXC6 among tomato GRX members.

After confirming the V2-SlGRXC6 interaction, we set out to identify the domain, motif, and amino acids in SlGRXC6 that were critical for the interaction. Based on a motif scan analysis (http://www.ebi.ac.uk/interpro/), there are two conserved domains in SlGRXC6. One domain is glutaredoxin PF00462, spanning amino acids 49 to 84 and the other is glutaredoxin PS51354, spanning amino acids 37 to 113 of SIGRXC6. We constructed seven SlGRXC6 deletion mutants as shown in Fig 1D, and determined that the shortest region interacting with V2 was SlGRXC6-M3 (aa 49–84) (Fig 1D).

To more specifically identify the amino acids in the M3 fragment that are involved in the interaction, we selected five amino acids based on post-translational modifications. We constructed mutants with a single alanine substitution in T53, G56, C58, S69, and S72 to make constructs SlGRXC6^T53A^, SlGRXC6^G56A^, SlGRXC6^C58A^, SlGRXC6^S69A^, and SlGRXC6^S72A^, respectively. Based on the Y2H analysis (Fig 1E), SlGRXC6^T53A^ and SlGRXC6^C58A^ failed to interact with V2, suggesting that T53 and C58 are possibly the key sites in SlGRXC6 for interaction with V2.

We then tested the interactions of SlGRXC6^T53A^ and SlGRXC6^C58A^ with V2 using the co-IP assay in plant cells. YFP-tagged WT SlGRXC6, SlGRXC6^T53A^, SlGRXC6^C58A^, or YFP were coexpressed with FLAG-V2, and the total lysate was subjected to the co-IP assay. Although WT or SlGRXC6 mutants accumulated to similar levels, only WT SlGRXC6-YFP, but not SlGRXC6^T53A^- or SlGRXC6^C58A^-YFP, was pulled down along with FLAG-V2 (Fig 1C). This confirmed that the Ala substitution in T53 or C58 affected the ability of SlGRXC6 to interact with V2.

We next tested whether such mutations affect localization of SlGRXC6. When SlGRXC6^T53A^- or SlGRXC6^C58A^-YFP was expressed in H2B transgenic *N*. *benthamiana*, the fluorescence signal of SlGRXC6^T53A^- or SlGRXC6^C58A^-YFP was similarly observed in the cytoplasm and nucleus in all cells (n = 30) (Fig 1F), indicating that the T53A or C58A substitution had no effect on localization of SlGRXC6. Western blotting confirmed that SlGRXC6^T53A^- or SlGRXC6^C58A^-YFP accumulated to a level similar to SlGRXC6 (Fig 1F), indicating that both mutations did not affect the expression and stability of mutant proteins.

### SlGRXC6 Regulates TYLCV Infection in Tomato Plants through Its Interaction with the V2 protein

**Fig 2:**
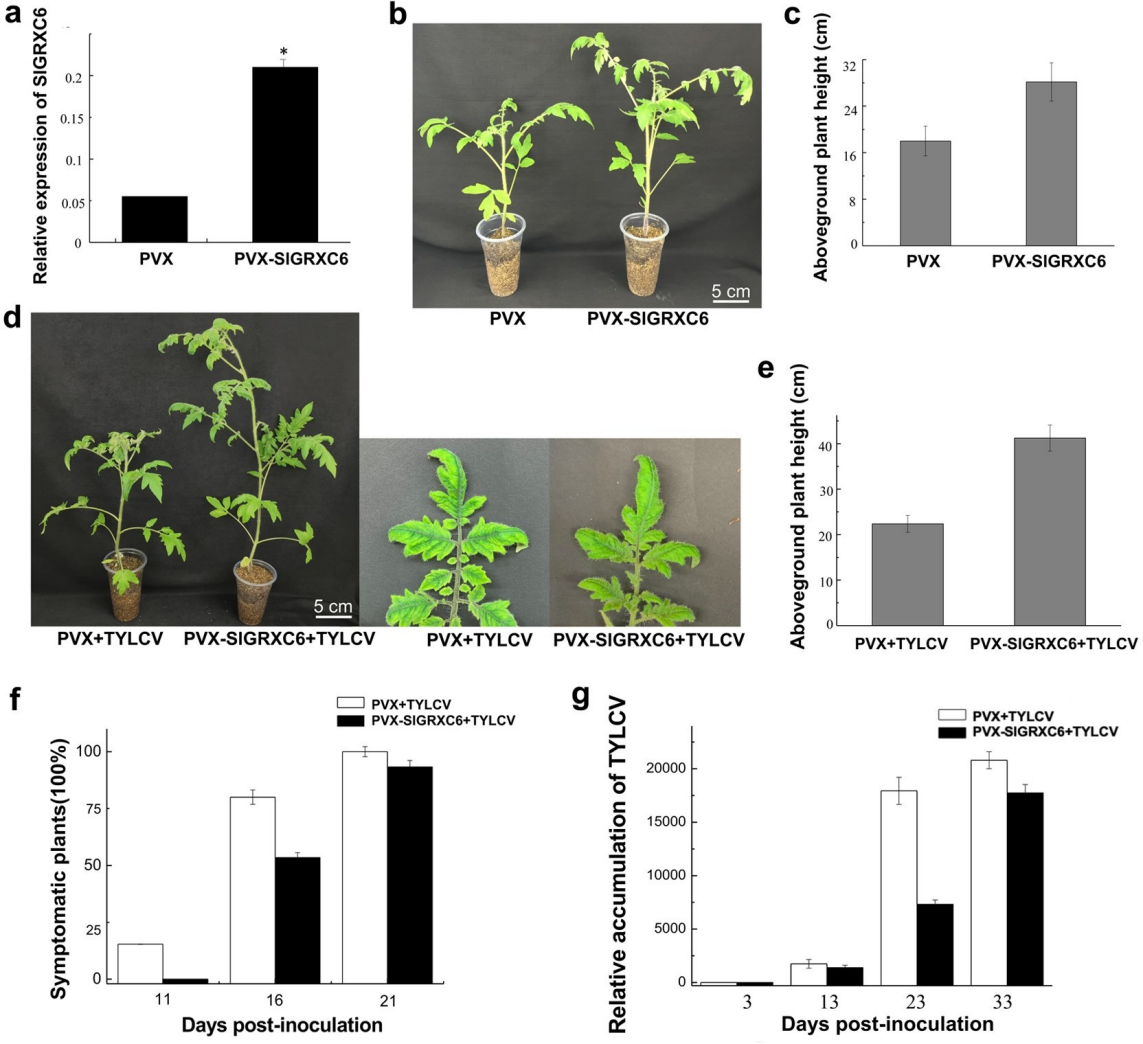
The overexpression of *SlGRXC6* inhibits TYLCV infection in tomato plants. (a) The relative levels of *SlGRXC6* transcripts in plants, which were treated with PVX or PVX-SlGRXC6, were determined by qRT-PCR at 8 dpai. Total RNA was extracted from newly emerged systemic leaves. Values represent the mean relative to the mock-treated plants (n = 3 biological replicates) and normalized with *SlActin* as an internal reference. Data are means ± SD (n = 3). Asterisk indicates a statistically significant difference (*p<0.05) according to Student’s t-test. (b) Tomato plants treated with PVX or PVX-SlGRXC6. Plants and newly emerged systemic leaves were photographed at 8 dpai. Bar: 5 cm. (c) The aboveground plant heights of PVX or PVX-SlGRXC6 plants were measured at 16 dpai. (d) Tomato plants treated with PVX and PVX-SlGRXC6 responded differently to TYLCV infection. Plants and newly emerged leaves were photographed 13 days after TYLCV inoculation. Bar: 5 cm. (e) The plant height of TYLCV-inoculated tomato plants treated with PVX or PVX-SlGRXC6. Plants were measured 13 days after TYLCV inoculation. (f) The time course of TYLCV infection in PVX control or PVX-SlGRXC6 plants. Values represent percentages of systemically infected plants at the indicated time points. In each experiment, 15 plants were inoculated and three independent repeats were performed. Experiments were repeated three times with similar results. (g) The viral genomic DNA accumulation in systemic leaves as measured by qPCR. Accumulated levels of viral genomic DNA were tested in PVX control or PVX-SlGRXC6 tomato plants infected with TYLCV at 3, 13, 23, and 33 dpi as in a. Data are means ± SD (n = 3). Experiments were repeated three times with similar results.

To assess the biological significance of the V2-SlGRXC6 interaction *in vivo*, we overexpressed *SlGRXC6* in tomato using a PVX vector [58]. The accumulated *SlGRXC6* transcripts in plants overexpressing *SlGRXC6* increased 3-fold more than that of PVX control plants at 8 days post-agroinfiltration (dpai), based on qRT-PCR (Fig 2A). Unexpectedly, the plants overexpressing *SlGRXC6* grew faster and taller than those with PVX only (Fig 2B). At 16 dpai, the average aboveground height of *SlGRXC6-*overexpressed plants was 28 cm, a 60% increase over that of PVX-inoculated plants (Fig 2C).

PVX- and PVX-SlGRXC6-treated plants were then infected with TYLCV at 8 dpai. At 13 days post-inoculation (dpi), TYLCV-infected PVX-SlGRXC6-plants showed milder symptoms compared to that of PVX-treated plants (Fig 2D). The aboveground height of *SlGRXC6*-overexpressed plants infected with TYLCV was much taller than that of WT plants (Fig 2E). At 11 days after TYLCV inoculation, 2 out of the 15 PVX-treated plants started showing symptoms, but all 15 PVX-SlGRXC6-treated plants remained symptomless (Fig 2F). Importantly, only 7 out of a total of 15 inoculated plants developed mild symptoms compared to 12 of the TYLCV-infected PVX plants, which had strong symptoms at 16 dpi (Fig 2F). Although all plants developed symptoms by 21 days, the TYLCV-infected PVX-SlGRXC6 plants showed weaker symptoms and in addition, at 23 dpi the accumulated TYLCV genomic DNA decreased by 57% compared to that of mock plants as revealed by qPCR (Fig 2G). However, accumulated viral DNA decreased 16% in PVX-SlGRXC6 plants compared to that in PVX plants by 33 dpi (Fig 2G). Given that the accumulated TYLCV genomic DNA in *SlGRXC6*-overexpressing plants was much lower at 23 dpi but similar at 33 dpi than those in mock plants, we conclude that overexpressed *SlGRXC6* can mitigate but cannot totally block TYLCV infection.

**Fig 3:**
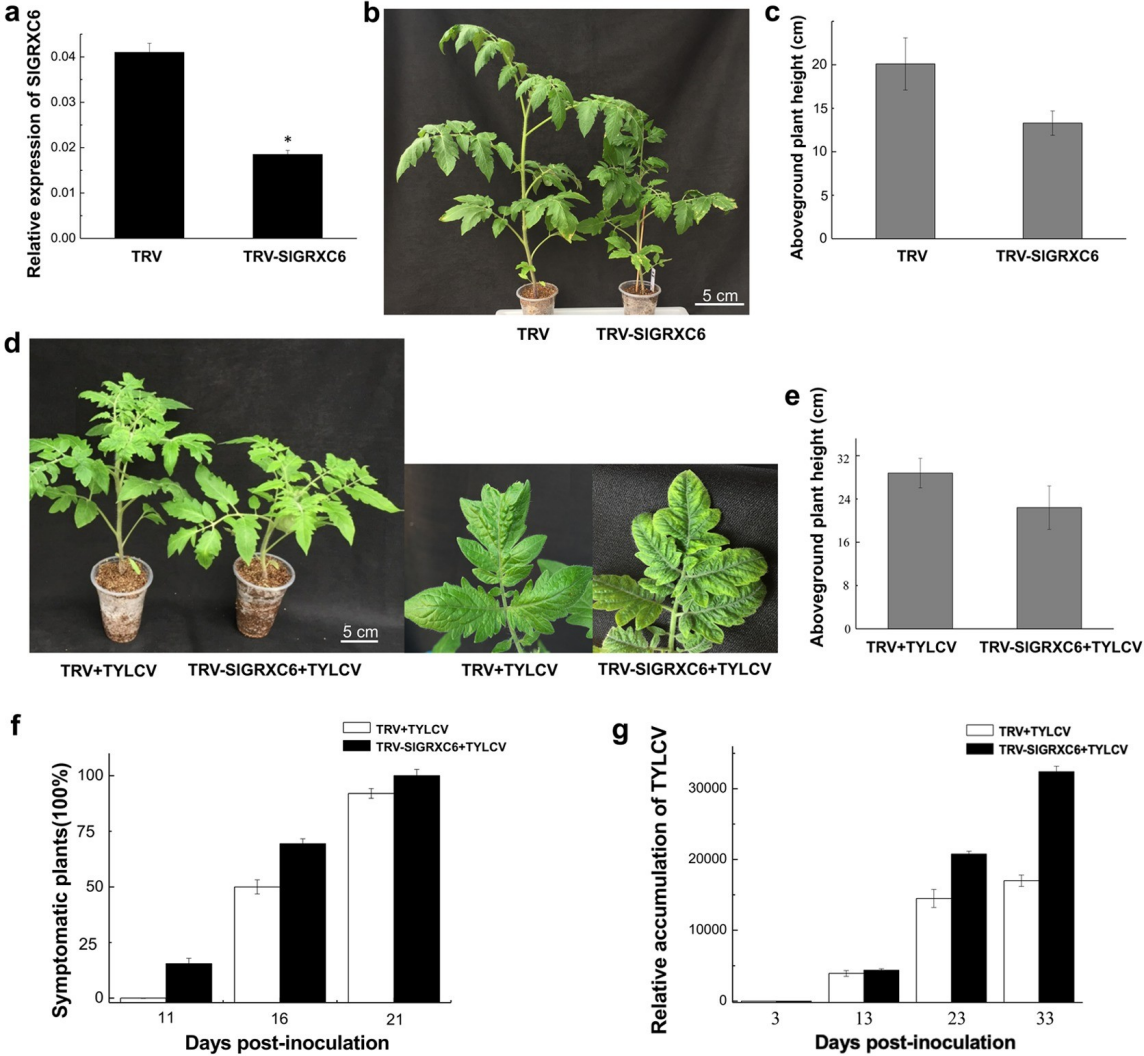
Knocking down the expression of *SlGRXC6* promotes TYLCV infection. (a) The relative levels of *SlGRXC6* transcripts in control (TRV) and knockdown (TRV-SlGRXC6) tomato plants were determined by qRT-PCR at 12 dpai with *SlActin* as an internal control. Data are means ± SD (n = 3). Asterisk indicates a statistically significant difference (*p<0.05) according to Student’s t-test. (b) Tomato plants are smaller when treated with TRV-SlGRXC6 compared to those treated with TRV. Bar: 5 cm. (c) The aboveground height of control and *SlGRXC6*-silenced plants were tested at 16 dpai. (d) Symptoms caused by TYLCV in control or *SlGRXC6*-silenced plants. Leaves were photographed at 13 dpi. Bar: 5 cm. (e) The aboveground height of TRV- or TRV-SlGRXC6-treated tomato plants as measured 13 days after TYLCV infection. (f) The time course of TYLCV infection in control or *SlGRXC6*-silenced plants as shown in Fig 2F. Experiments were repeated three times with similar results. (g) The accumulated viral genomic DNA in systemic leaves as measured by qPCR. Accumulated levels of viral genomic DNA were tested in control or *SlGRXC6*-silenced tomato plants infected with TYLCV at 3, 13, 23, and 33 dpi as shown in Fig 2A. Data are means ± SD (n = 3). Experiments were repeated three times with similar results.

We further tested TYLCV infection in *SlGRXC6*-silenced tomato plants. To knock down *SlGRXC6* expression, we used a virus-induced gene silencing (VIGS) approach that was mediated by *Tobacco rattle virus* (TRV) [59]. We used the endogenous phytoene desaturase gene (PDS) as a reporter for monitoring the effectiveness and progress of TRV-induced gene silencing. The upper leaves of TRV-PDS-infiltrated plants turned white at 12 dpai and the accumulated *SlPDS* transcripts were about 30% of those in TRV-infected leaf tissues (S3 Fig), similar to that reported in tomato plants [59]. Following the same protocol, we next inoculated tomato plants with TRV-SlGRXC6. The expression of *SlGRXC6* was downregulated in the *SlGRXC6-*silenced lines to 50% of that in the TRV control lines at 12 dpai (Fig 3A). In sharp contrast to the promoted plant growth in the *SlGRXC6-*overexpressing lines (Fig 2B), TRV-SlGRXC6-inoculated plants were shorter than TRV-inoculated plants (Fig 3B). At 16 dpai, the height of TRV-SlGRXC6 plants was 12 cm, significantly shorter than that of TRV-treated plants at 20 cm (Fig 3C).

TRV and TRV-SlGRXC6 plants were then inoculated with TYLCV at 12 dpai. The TRV-SlGRXC6 plants developed more severe symptoms and were much shorter than those of TYLCV-infected TRV plants (Fig 3D and 3E). At 11 days post TYLCV inoculation, 3 TRV-SlGRXC6 plants out of 15 started showing symptoms, but all 15 TRV plants remained symptomless (Fig 3F). At 16 dpi, there were more *SlGRXC6*-silenced plants showing strong symptoms with 11 out of 15 plants developing typical symptoms, compared to 8 out of 15 of the TYLCV-infected mock plants showing symptoms (Fig 3F). Although almost all plants had symptoms by 21 days post-TYLCV infection, TRV-SlGRXC6 plants showed more severe symptoms compared to TRV plants. Moreover, viral DNA accumulated at greater levels in *SlGRXC6*-silenced plants than in mock-inoculated plants (Fig 3G), suggesting that SlGRXC6 restricts TYLCV infection.

**Fig 4:**
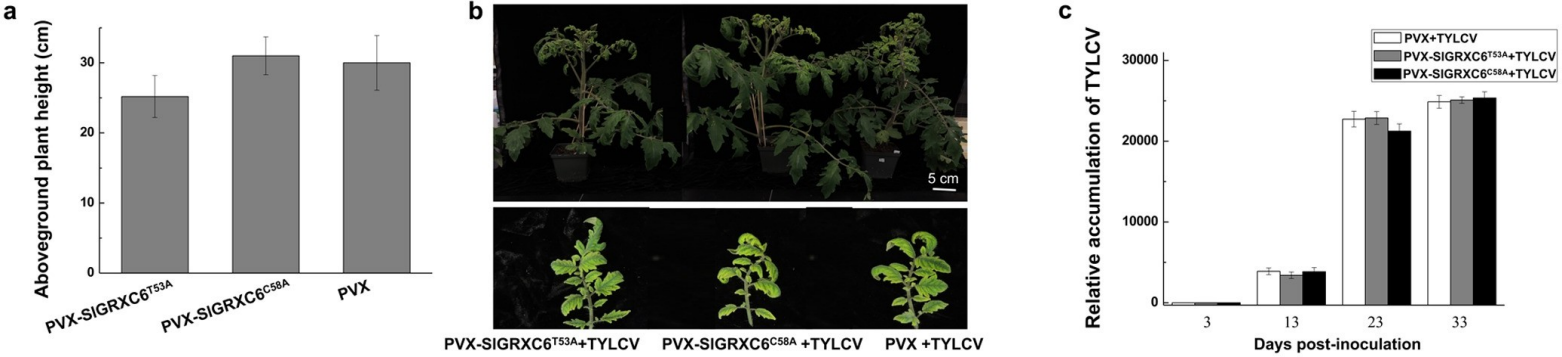
The V2-SlGRXC6 interaction is critical to TYLCV infection. (a) The aboveground height of PVX-, PVX-SlGRXC6^T53A^-, or PVX-SlGRXC6^C58A^-inoculated tomato plants as measured at 16 dpai. (b) Symptoms in control, PVX-SlGRXC6^T53A^-, PVX-SlGRXC6^C58A^-inoculated plants. Leaves were photographed 23 days after TYLCV inoculation. Bar: 5 cm. (c) The accumulated TYLCV viral DNA in systemic leaves as measured by qPCR. Accumulated levels of viral genomic DNA were tested in PVX-, PVX-SlGRXC6^T53A^-, PVX-SlGRXC6^C58A^-treated tomato plants infected with TYLCV at 3, 13, 23, and 33 dpi as in Fig 2A. Data are means ± SD (n = 3). Experiments were repeated three times with similar results.

To further verify the effect of V2-SlGRXC6 interaction on viral infection, we overexpressed *SlGRXC6*^*T53A*^ or *SlGRXC6*^*C58A*^ in tomato plants using the PVX vector. The aboveground plant height of SlGRXC6^T53A^- and SlGRXC6^C58A^-overexpressed plants were similar to that of mock plants (Fig 4A). Moreover, the symptoms and viral DNA accumulated at similar levels in all TYLCV-inoculated plants (Fig 4B and 4C). Given the fact that both mutants accumulated at levels similar to WT (Fig 1F), these data showed that SlGRXC6 mutations had no effect on viral infection, suggesting that SlGRXC6 regulates TYLCV infection possibly through its interaction with V2 in tomato plants.

To rule out the possibility that the inhibited TYLCV infection in TRV-SlGRXC6-treated plants was not due to the slowed plant growth, we included three other different tomato GRXs that were from the same CC-type group as SlGRXC6, SlGRX25, SlGRX38, and SlGRX39. SlGRX25 and SlGRX38 did not interact but SlGRX39 weakly interacted with V2 (S2B Fig). Approximately 70% decrease of accumulated transcripts of *SlGRXC6*, *SlGRX25*, *SlGRX38*, or *SlGRX39* was achieved by using TRV-mediated VIGS. All TRV-SlGRXC6, -SlGRX25, -SlGRX38, or -SlGRX39-inoculated plants were shorter than TRV-inoculated plants (S4A, S4B and S4C Fig), confirming that the CC-type GRXs are related to plant growth [36]. Interestingly, the accumulated viral DNA levels in *SlGRX25*, *SlGRX38*, or *SlGRX39*-silenced plants were similar to those in the TRV-treated plants (S4D and S4E Fig). On the contrary, TRV-SlGRXC6 plants developed more severe symptoms and accumulated higher levels of viral DNA (S4D and S4E Fig), indicating that plant growth and TYLCV viral susceptibility are not coupled.

### SlGRXC6 Inhibits the Nuclear Export of the V2 Protein

**Fig 5:**
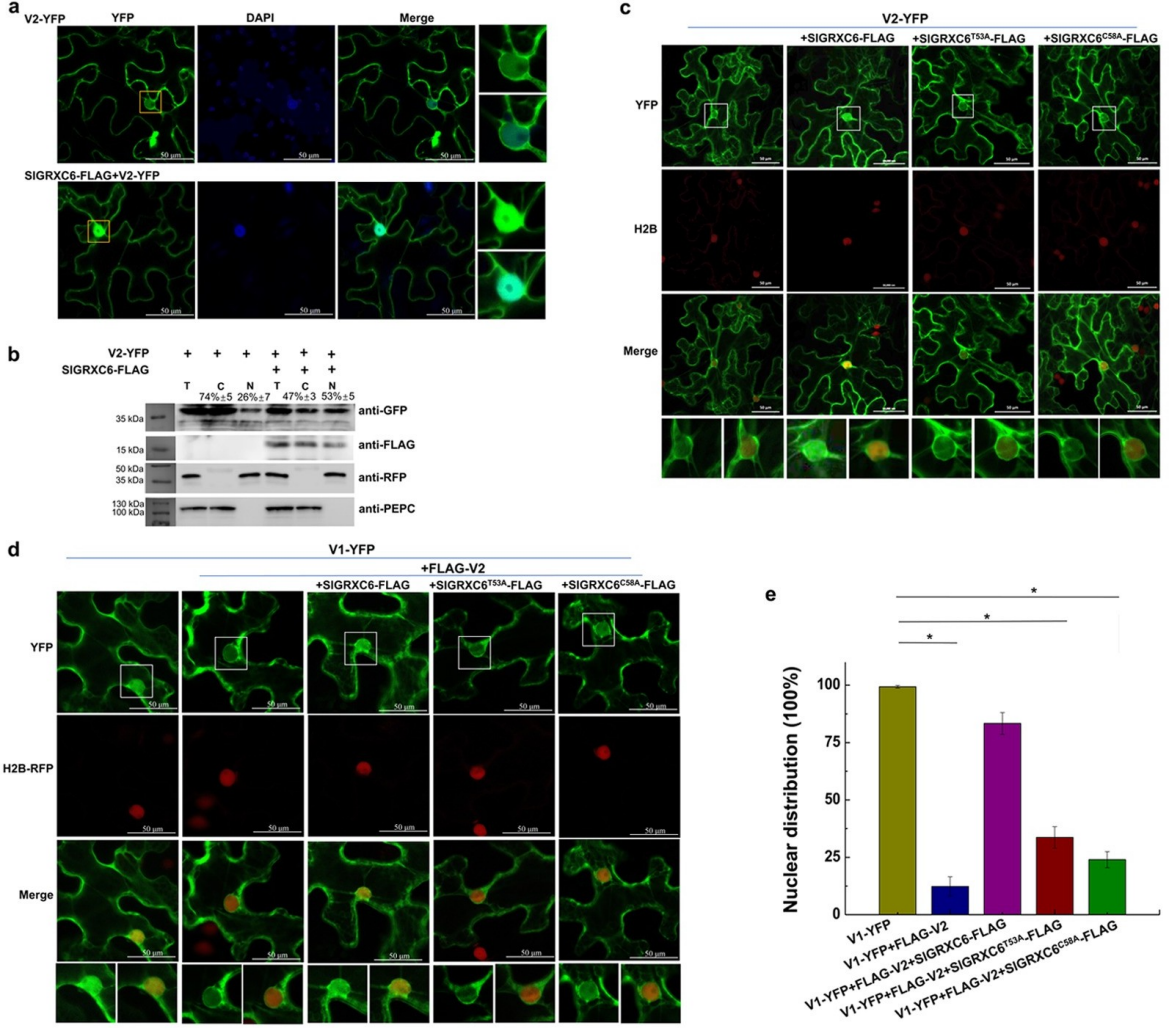
The effect of SlGRXC6 on the nuclear distribution of the V2 and V1 protein. (a) Localization of V2-YFP in the absence or presence of the SlGRXC6 protein in *N*. *benthamiana* cells. V2-YFP expressed alone or coexpressed with SlGRXC6-FLAG was detected by confocal microscopy. Experiments were repeated three times and 30 cells were observed in each repeat. DAPI stains DNA in the nucleus. Bars: 50 μm. (b) The distribution of V2 in the absence or presence of SlGRXC6-FLAG in H2B-RFP transgenic *N*. *benthamiana* plants was analyzed using a nuclear-cytoplasmic fractionation assay. Western blotting was conducted with antibodies specific to the indicated proteins. PEPC and H2B-RFP were used as markers for the cytoplasmic and nuclear fraction, respectively. The intensity of the protein signal was measured using ImageQuant TL (GE healthcare), with levels of the cytoplasm and nucleus totaling 100%. Values represent the average of three plants. Experiments were repeated three times. (c) Localization of V2-YFP when expressed alone or in the presence of SlGRXC6, SlGRXC6^T53A^, or SlGRXC6^C58A^ in H2B transgenic *N*. *benthamiana* cells. Bars: 50 μm. Experiments were repeated three times and 30 cells were observed in each repeat. (d) Subcellular localization of V1 was expressed alone or coexpressed with FLAG-V2 and FLAG-tagged SlGRXC6, SlGRXC6^T53A^, or SlGRXC6^C58A^ in H2B transgenic *N*. *benthamiana* cells. The H2B-RFP signal represents the nucleus. The enlarged areas show the nuclear region. Bars: 50 μm. Experiments were repeated three times and 30 cells were observed in each repeat. (e) The number of cells with a nuclear distribution of V1-YFP was counted and the percentage of cells with a nuclear distribution was calculated. Experiments were repeated three times and 30 cells were observed in each repeat. Values represent percentages of cells with nuclear distribution of YFP signal ± SD (standard deviation). Data were analyzed using Student’s t-test and asterisks denote significant differences (*P < 0.05).

We previously reported that the fluorescence signal was observed in the cytoplasm and the perinuclear region in cells expressing V2-YFP [60]. However, the V2-SlGRXC6 complex was observed in the nucleus and cytoplasm (Fig 1B), suggesting that SlGRXC6 may retain V2 in the nucleus. We then tested the effect of SlGRXC6 on the localization of V2. When FLAG-tagged SlGRXC6 (FLAG-SlGRXC6) and V2-YFP were coexpressed in *N*. *benthamiana* cells, strikingly, a very strong V2-YFP fluorescence signal was found in the nucleus in almost all the cells (n = 30) (Fig 5A), showing that SlGRXC6 can enhance nuclear accumulation of V2. We also coexpressed V2-RFP with SlGRXC6-YFP and found that V2-RFP was present in the nucleus and the cytoplasm (n = 20) (S5 Fig). These results suggest that SlGRXC6 inhibits the nuclear export of V2, increasing its accumulation in the nucleus.

To complement our visual observations with a biochemical assay, we performed a fractionation assay to separate total lysate into its nucleus and cytoplasm fractions [61]. As shown in Fig 5B, we only detected the cytoplasmic marker phosphoenolpyruvate carboxylase (PEPC) in the cytoplasmic fraction, and the nuclear marker H2B-RFP was only present in the nuclear fraction. Under such conditions, the amount of V2-YFP in the nuclear fraction significantly increased in the presence of SlGRXC6-FLAG, which is consistent with the results based on fluorescence microscopy (Figs 5A and S5). To provide numeric readings, we set the sum of the V2-YFP signal intensity in the cytoplasm and nucleus at 100%. In the presence of SlGRXC6-FLAG, we found 53% of V2-YFP in the nuclear fraction compared to 26% when V2-YFP was expressed alone (Fig 5B). This was in sharp contrast to H2B-RFP and PEPC whose localizations were not affected in the presence of SlGRX6-FLAG. According to our early results, V2 depends on exportin-⍺ to exit from the nucleus [60]; thus, we concluded that SlGRXC6 is able to enrich V2 in the nucleus, possibly by preventing V2 from leaving the nucleus.

We next tested whether the V2-SlGRXC6 interaction is required for SlGRXC6-mediated enrichment of V2 in the nucleus. When coexpressed with either SlGRXC6^T53A^ or SlGRXC6^C58A^, V2-YFP was primarily found in the cytoplasm and the perinuclear region in most cells (n = 30), but not in the nucleus (Fig 5C), suggesting that T53 and C58 are critical for SlGRXC6 to re-localize V2. Because both mutants accumulated at a similar level to WT SlGRXC6 (Fig 1F), these results showed that SlGRXC6 inhibited the nuclear export of V2 through its interaction with V2.

We previously showed that V2 facilitates the nuclear export of TYLCV V1 protein [60]. As shown in Fig 5D and 5E, V1-YFP was found in the nucleus in all the cells (n = 30) when expressed alone but in only 17% cells (n = 30) in the presence of FLAG-V2. Given that SlGRXC6 enhanced the nuclear accumulation of V2 (Fig 5A and 5B), it is possible that SlGRXC6 may prevent the nuclear egress of V1 via the inhibited V2 export. Agreeing with this notion, V1-YFP was detected in the nucleus in 86% of cells (n = 30) when coexpressed with FLAG-V2 and SlGRXC6-FLAG (Fig 5D and 5E). We also tested whether SlGRXC6 executed its functions via its interaction with V2 by using mutants SlGRXC6^T53A^ and SlGRXC6^C58A^. When co-expressed with FLAG-V2 and SlGRXC6^T53A^- or SlGRXC6^C58A^-FLAG, only 24–33% of cells had V1-YFP in the nucleus (Fig 5D and 5E), suggesting that the SlGRXC6-V2 interaction is necessary to inhibit the V2-mediated nuclear egress of V1.

Based on earlier reports that V1 is involved in binding and nuclear shuttling of viral genomic DNA [22,62], we hypothesized that SlGRXC6 may restrict TYLCV infection by inhibiting the nuclear export of the V1-viral genomic DNA complex. We measured accumulated TYLCV genomic DNA in the nucleus in TYLCV-inoculated SlGRXC6-overexpressed and -silenced tomato plants. We harvested emerging leaves from TYLCV-inoculated plants and extracted DNA from the total lysate or the nuclear fraction separately. As shown in S6 Fig, in the nuclear fraction we only detected proliferating cell nuclear antigen (PCNA) but not the cytochrome C oxidase subunit 1 (COX1, locus ATMG01360), which is the mitochondrial catalytic-core subunit of the respiratory chain complex and is encoded by mitochondrial DNA [63]. Under such conditions, viral DNA levels in the nuclear fraction and total lysates were detected by qPCR and the percentages of accumulated TYLCV genomic DNA in the nuclear fraction compared to that of total lysates were calculated. A significant difference in the nucleus-accumulated viral DNA was observed in SlGRXC6-overexpressed plants compared to that of control plants at 23 dpi: 14.4% of viral DNA accumulated in the nucleus in SlGRXC6-overexpressed plants compared to 4.85% in the control plants (S7A Fig). These results showed that SlGRXC6 inhibited the viral DNA export from the nucleus during the period of high levels of DNA replication, likely by inhibiting V2 export. Conversely, as shown in S7B Fig, statistical analysis showed that the accumulated viral DNA in the nucleus was significantly reduced in SlGRXC6-silenced plants at 23 dpi. In addition, overexpression of SlGRXC6^T53A^- or SlGRXC6^C58A^ failed to inhibit viral infection (Fig 4), showing that the SlGRXC6-V2 interaction is necessary for inhibiting viral infection. These results suggested that the V2-SlGRXC6 interaction is required for the inhibited nuclear egress of V2 and V1, as well as TYLCV infection.

We also tested whether SlGRXC6 restricted TYLCV by affecting gene expression of pathogenesis-related (PR) genes. As shown in S8 Fig, accumulated transcripts of *PR1-a*, *GLUA* and *CHI3*, three tomato PR genes [64], decreased by 42%-58% in TYLCV-inoculated, TRV-SlGRXC6 plants compared to those in the TYLCV-infected TRV plants (S8 Fig). Knocking down gene expression of *SlGRX38* or *SlGRX39* also decreased the accumulated PR gene transcripts similarly to that *SlGRXC6* (S8 Fig), however, only the knockdown of *SlGRXC6* inhibited TYLCV infection but not *SlGRX38* and *SlGRX39* (S4 Fig), suggesting that the enhanced nuclear egress of V2, but not the decreased expression of PR genes, is the major force contributing to the promoted TYLCV infection in the *SlGRXC6*-silenced tomato plants.

### SlGRXC6 Mediates Plant Growth along with SlNTRC80

**Fig 6:**
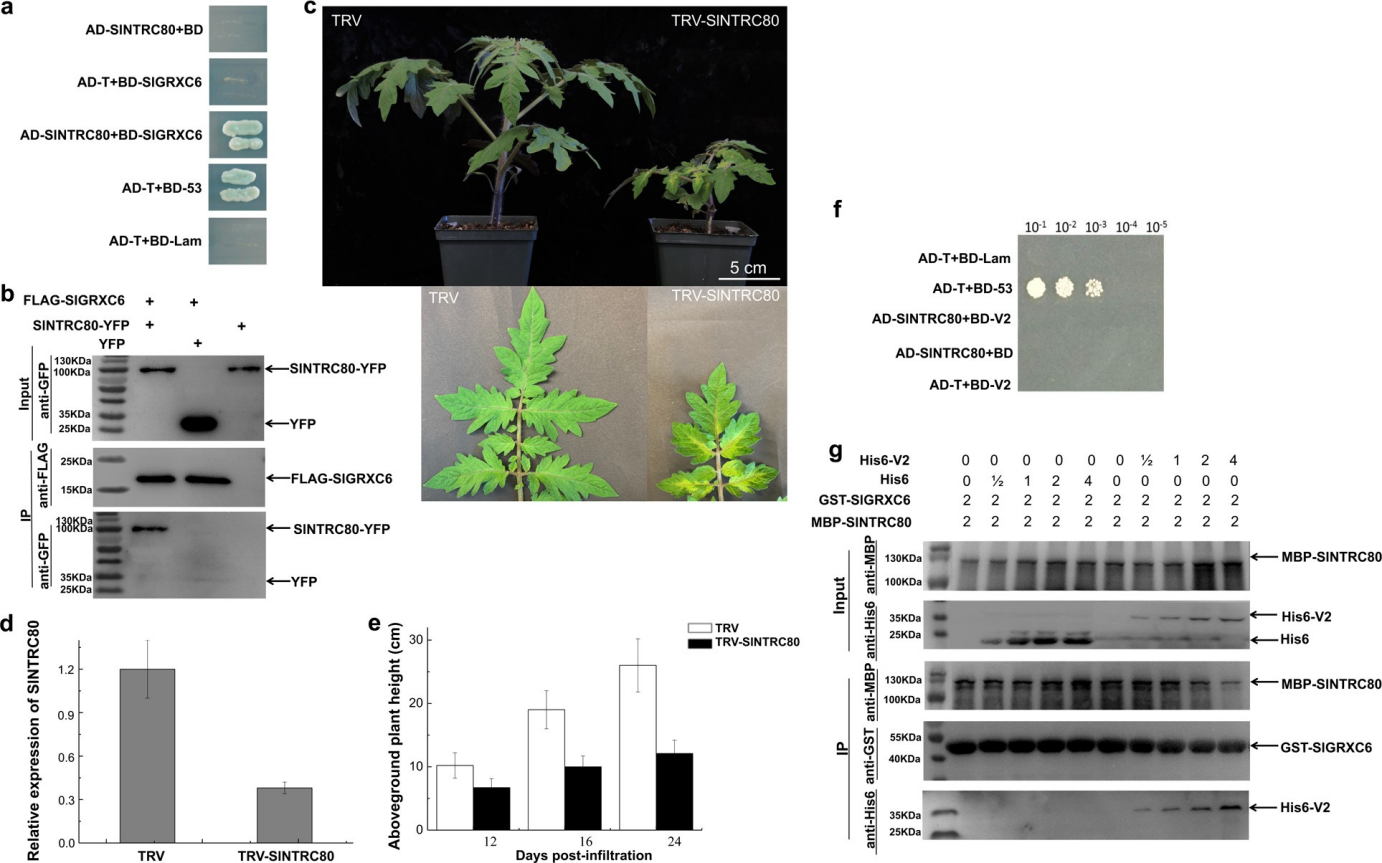
Identification of the interaction between SlGRXC6 and SlNTRC80. (a) The interaction between SlGRXC6 and SlNTRC80 was tested in the Y2H. Yeast cells expressing the indicated pairs of proteins grew on selection medium (SD/- His/-Leu/-Trp/-Ade) supplied with X-α-Gal. (b) The SlGRXC6-SlNTRC80 interaction was confirmed by co-IP assay. (c) Tomato plants treated with TRV or TRV-SlNTRC80. Plants and newly emerged systemic leaves were photographed at 12 dpai. Bar: 5 cm. Experiments were repeated three times with similar results. (d) Relative expression of *SlNTRC80* was tested in control and *SlNTRC80*-silenced plants using qRT-PCR as shown in Fig 2A. Data are means ± SD (n = 3). (e) Aboveground plant heights of control and *SlNTRC80*-silenced plants at 12, 16, and 24 dpai. (f) The interaction between V2 and SlNTRC80 was tested in Y2H. Yeast cells expressing the indicated pair of proteins were plated onto the selection medium (SD/-His/-Leu/-Trp) in 10-fold serial dilutions. (g) *In vitro* competitive pulldown assays. The indicated amounts of His6-V2 or His6 protein were mixed with 2 μg of MBP-SlNTRC80 and pulled down by 2 μg of GST-SlGRXC6. The bound protein was detected by immunoblotting with the indicated antibodies. Experiments were repeated three times with similar results.

It is well-known that GRXs function together with TRX in plant development, stress responses, and host-mediated pathogen responses [45,47,48]. Given the fact that the levels of SlGRXC6 are related to plant growth (Figs 2B and 3B), we hypothesized that the stunting symptom induced by TYLCV infection may be related to SlGRXC6 directly, or indirectly via SlGRXC6-interacting TRX(s). Based on our analysis using the STRING program (https://string-db.org/), 10 tomato TRXs were predicted to be associated with SlGRXC6 (S9A Fig). Among them, only SlTRX1-140 (Thioredoxin like 1–140) and SlNTRC80 (NADPH-dependent thioredoxin reductase C80) were found to interact with SlGRXC6 in the Y2H (Figs 6A and S9B). SlGRXC6-SlNTRC80 and SlGRXC6-SlTRX1-140 interactions were further tested by the co-IP assay. However, only FLAG-SlNTRC80 was detected when SlGRXC6-YFP was pulled down using an anti-GFP polyclonal antibody (Fig 6B).

Although it is well-known that NTRs play an important role in plant growth [51,54], the possible role of SlNTRC80 and its interaction with other GRXs is unknown. We knocked down the expression of *SlNTRC80* using the TRV vector. A strong growth defect was observed in *SlNTRC80*-silenced plants, including yellowish young leaves and dwarf plants (Fig 6C, 6D and 6E). These results agreed with previous studies in *Arabidopsis* [51–53], suggesting that SlNTRC80’s role in plant growth that might be conserved among multiple plant species.

We next examined the subcellular localization of SlNTRC80-YFP in H2B-RFP transgenic *N*. *benthamiana* cells. The fluorescence of SlNTRC80-YFP was localized in the cytoplasm and nucleus in all cells (n = 20) (S10 Fig). We next tested the effect of *SlGRXC6* on the expression of *SlNTRC80*. An up-regulation of *SlNTRC80* expression was found in tomato plants when SlGRXC6 was overexpressed using PVX as a vector (S11A Fig), and a decreased level of *SlNTRC80* was found in TRV-SlGRXC6-infiltrated tomato plants (S11B Fig). The fact that the levels of SlGRXC6 correlated well with those of SlNTRC80 suggest that they may be coordinately involved in plant growth.

We also tested whether the SlGRXC6-SlNTRC80 interaction is necessary for growth regulation. We first tested whether SlGRXC6^T53A^- and SlGRXC6^C58A^-FLAG interacted with SlNTRC80-YFP using the co-IP assay. As shown in S12 Fig, only a very weak band of SlNTRC80-YFP was detected when FLAG-SlGRXC6^C58A^ and SlNTRC80 were coexpressed. No SlNTRC80-YFP was pulled down when they are co-expressed with SlGRXC6^T53A^-FLAG, showing that SlGRXC6^T53A^ and SlGRXC6^C58A^ lost the ability to interact with SlNTRC80. In addition, SlGRXC6^T53A^- or SlGRXC6^C58A^-overexpressed tomato plants were not significantly different from the control plants (Fig 4A and 4B), suggesting that SlGRXC6 and SlNTRC80 may work together to regulate the growth of tomato plants.

### V2 Competes with SlNTRC80 for Direct Binding to SlGRXC6

We next tested whether V2 interacted with SlNTRC80. As shown in Fig 6F, no interaction between SlNTRC80 and V2 was observed in Y2H. Given that SlGRXC6 functioned as a restriction factor (Figs 2 and 3) and TYLCV infection promoted the expression of *SlGRXC6* (S13 Fig), we hypothesized that SlGRXC6 and SlNTRC80 may function as a complex in regulating plant growth but that SlGRXC6 may be recruited away or sequestered by V2 during TYLCV infection. To test the possibility that V2 may compete with SlNTRC80 for binding with SlGRXC6, we performed a competitive pulldown assay using glutathione S-transferase (GST)-tagged SlGRXC6 (GST-SlGRXC6), maltose binding protein (MBP)-tagged SlNTRC80 (MBP-SlNTRC80), and His6-tagged V2 (His6-V2) that were purified from *E*. *coli*. Different amounts of His6-V2 or His6 were mixed with the same amount (2 μg) of GST-SlGRXC6 and incubated with GST-beads for 1 hour. Then, 2 μg MBP-SlNTRC80 were added and incubated with the beads. After extensive washing, GST-beads were collected and proteins that were pulled down with the beads were detected using anti-His6, -MBP, or -GST antibody. We found that the amount of MBP-SlNTRC80 pulled down by GST-SlGRXC6 was reduced when increasing amounts of V2 were added to the GST-SlGRXC6 and the MBP-SlNTRC80 mixture, but not by His6 (Fig 6G), suggesting that V2 has a higher affinity to SlGRXC6 than SlNTRC80 and possibly recruits SlGRXC6 away from SlNTRC80.

## Discussion

Geminiviruses infect a broad spectrum of plants and induce a wide range of symptoms. Some early reports showed that V2 of geminiviruses is an important symptom determinant [6,12–14, 16,19,29]. TYLCV is a major tomato pathogen worldwide that causes extensive tomato losses [65]. We found that TYLCV V2 caused yellowing and curly leaves, as well as dwarfness when expressed using a PVX vector in *N*. *benthamiana* [29] or expressed stably in transgenic tomato plants (S1 Fig), indicating that V2 plays a very important role in symptom development during TYLCV systemic infection. TYLCV V2 is a multifunctional protein and executes its functions along with many of its host partners. V2 inhibits TGS by interacting with host histone deacetylase 6 (HDA6), resulting in the decreased methylation of viral DNA [20]. V2 interacts with the cellular suppressor of gene silencing 3 (SGS3), which is required in the RNA silencing pathway, to suppress host RNA silencing [18]. V2 also interacts with CYP1, a tomato papain-like cysteine protease that is involved in plant defence against diverse pathogens [21]. To identify the host factors that might be involved in viral infection and symptom development along with V2, we identified SlGRXC6, out of 55 available GRXs in tomato, as a specific V2-interacting protein. We further demonstrated that SlGRXC6 possibly plays two roles in TYLCV infection: SlGRXC6 functions as a restriction factor of TYLCV that prevents V2 from moving out of the nucleus (Fig 5A and 5B), and in turn, the nuclear export of V1 (Fig 5D and 5E) and viral genomic DNA (S7 Fig). SlGRXC6 also contributes to symptom development (Figs 2 and 3) via its interaction with SlNTRC80 (Fig 6A and 6B), where V2 sequesters SlGRXC6 away from forming the SlGRXC6-SlNTRC80 complex (Fig 6G), and thus, inhibits plant growth (Fig 4). Our work, therefore, identified a new host partner of V2, and revealed the mechanisms whereby V2 functions as a pathogenicity determinant and can be targeted for antiviral defense.

For the role of GRXs in plant defense response, most reports focus on the enhanced generation of reactive oxygen species (ROS) that mediates plant defense gene activation and/or conjuncts with other plant signaling molecules, and thus, regulating pathogen infection, such as fungi and bacteria [47,66–68]. However, little has been reported on the interaction between plant viruses and host GRXs. We demonstrated that V2-YFP was localized in the cytoplasm and perinuclear region, but was highly enriched in the nucleus in the presence of SlGRXC6-FLAG in *N*. *benthamiana* cells (Fig 5A), suggesting a possible role of SlGRXC6 in restricting V2 from exporting out of the nucleus. Agreeing with prior reports that V2 facilitates the nuclear export of V1 [60] and V1 is involved in the nuclear shuttling of viral genomic DNA [22,62], we showed that V1 was also enriched in the nucleus in the presence of SlGRXC6 and V2 (Fig 5D and 5E) and the accumulated viral genomic DNA in the nucleus increased in the presence of overexpressed SlGRXC6 but decreased when the SlGRXC6 expression was knocked down (S7 Fig). Our data also demonstrated that SlGRXC6 executed its functions via its interaction with V2 based on two mutations in SlGRXC6, T53A and C58A, that disrupted the V2-SlGRXC6 interaction (Fig 1C and 1E). When coexpressed with SlGRXC6^T53A^- or SlGRXC6^C58A^-FLAG, V2-YFP localized in the perinuclear region and the cytoplasm (Fig 5C); when coexpressed with FLAG-V2 and SlGRXC6^T53A^- or SlGRXC6^C58A^-FLAG, only 24–33% of V1-YFP in the nucleus (Fig 5D and 5E), compared to 86% of cells with V1-YFP in the nucleus when co-expressed with FLAG-V2 and WT SlGRXC6, suggesting that the SlGRXC6-V2 interaction is necessary to inhibit the V2-mediated nuclear export of V1. In addition, we found that overexpression of SlGRXC6^T53A^- or SlGRXC6^C58A^ failed to inhibit viral infection (Fig 4), showing that the SlGRXC6-V2 interaction is necessary for inhibiting viral infection. These results suggested that SlGRXC6 functions as a host restriction factor for TYLCV infection by regulating the viral nuclear export process: SlGRXC6 interacts and inhibits the nuclear export of V2, and thus, the V2-mediated nuclear export of V1 and viral genomic DNA, and therefore TYLCV systemic infection.

In plants, GRXs and TRXs play key roles in the maintenance of cellular redox homeostasis, development, and responses to biotic or abiotic stresses [34,36,55,69]. In addition, the glutathione pathway compensates for the reduced NTR activity, suggesting an interplay between the TRX and GRX pathways [55,69]. It was reported that in transgenic *Arabidopsis*, the overexpression of NTR enlarged plants, but the lack of NTR led to smaller plants [51–53]. However, it is unknown whether the functions of NTR in regulating plant growth is conserved among different plant species. Our data showed that tomato plants were taller than control plants in the presence of overexpressed SlGRXC6 (Fig 2B) but became shorter when SlGRXC6 was down-regulated (Fig 3B), suggesting that GRX(s) have a similar function in plant growth. In addition, plants with silenced SlNTRC80 were smaller than control plants (Fig 6C), indicating that NTR from different plant species share similar functions in regulating plant growth. Our data support the notion that SlGRXC6 and SlNTRC80 work coordinately in regulating plant growth because SlGRXC6 interacted with SlNTRC80 (Fig 6A and 6B) and an increased level of SlNTRC80 was associated with the overexpressed SlGRXC6 (S11 Fig). Further supporting this notion, the overexpression of SlGRXC6^T53A^ or SlGRXC6^C58A^ failed to stimulate plant growth (Fig 4) and this failed stimulation was most likely related to the disrupted SlGRXC6-SlNTRC80 interaction (S12 Fig).

Several reports have shown that GRXs play an important role in host defense response [34,37]. We found that SlGRXC6 interacted with both V2, to restrict viral infection and virus-caused disease symptoms, and SlNTRC80, to regulate plant growth. Our data suggested that V2 and SlNTRC80 might bind to the same site in SlGRXC6 because in an *in vitro* competitive pull-down assay (Fig 6G), an increasing amount of V2 competed off SlNTRC80, suggesting V2 may have a higher affinity to SlGRXC6 than to SlNTRC80. Furthermore, SlGRXC6^T53A^ and SlGRXC6^C58A^ are defective in interacting with V2 (Fig 1C and 1E) and also SlNTRC80 (S12 Fig). Both mutants accumulated to the same levels to WT, suggesting that both were well-expressed and stable, even though we could not totally rule out that each mutation cause other defects in SlGRXC6.

**Fig 7:**
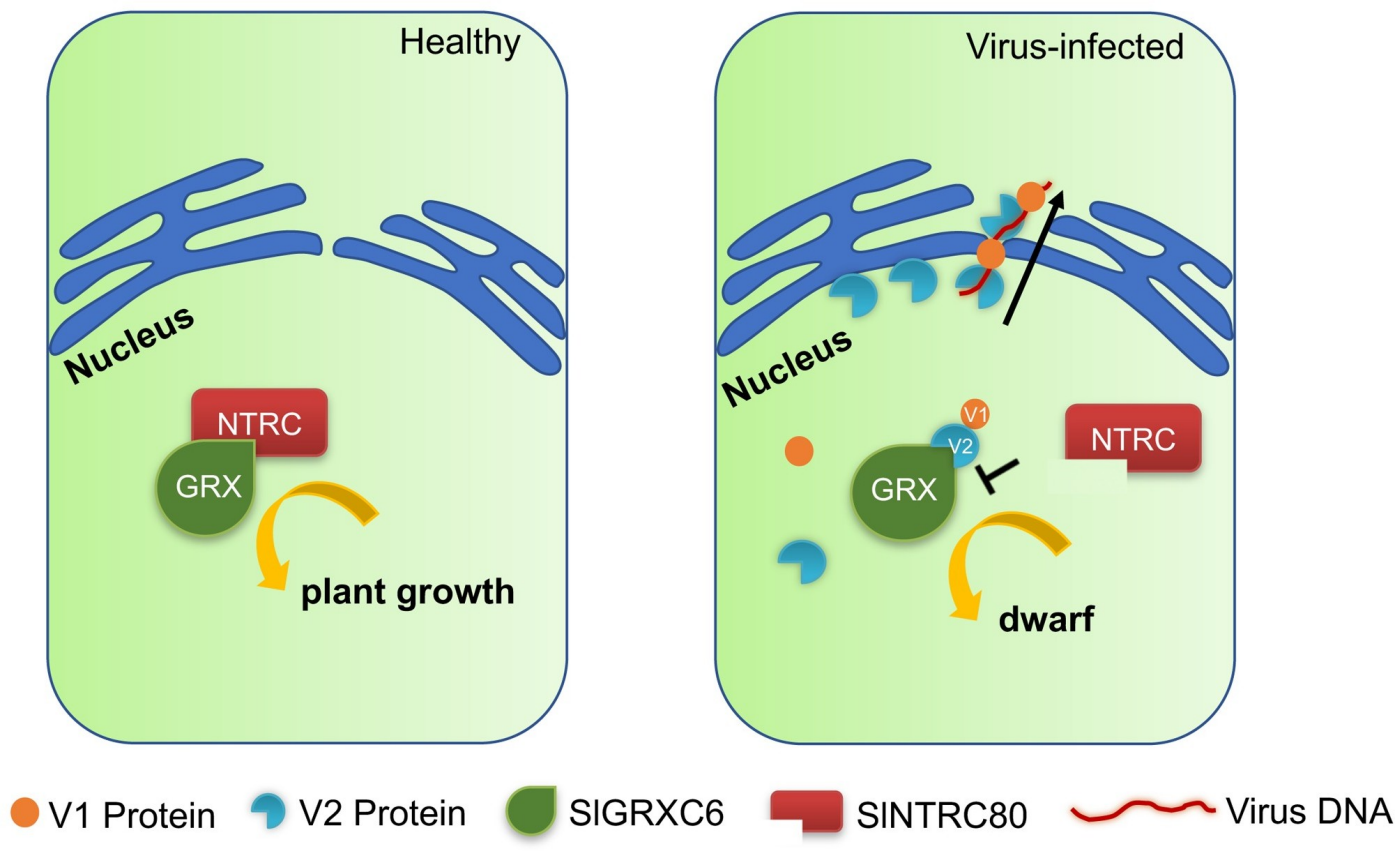
A proposed model for possible roles of the SlGRXC6-V2 interaction in the regulation of plant growth and the restriction of TYLCV infection. SlGRXC6 and SlNTRC80 physically interact and are cooperatively involved in regulating plant growth. Disruption of the interactions or the balance between them leads to growth defects. The TYLCV V2 protein is a symptom determinant and interacts with SlGRXC6 during viral infection. The V2-SlGRXC6 interaction inhibits or slows down the nuclear export of V2, the V2-mediated nuclear export of V1, and the V1-viral DNA complex, and therefore, restricts viral infection. The V2-SlGRXC6 interaction also sequesters SlGRXC6 from binding to SlNTRC80, interfering the SlNTRC80-SlGRXC6 interaction and leading to growth defects.

Based on our work and results from others, we propose a working model underlying a possible mechanism by which SlGRXC6 restricts viral infection and contributes to symptom development (Fig 7). SlGRXC6 restricts TYLCV infection by preventing V2 from moving out of the nucleus, and thus, inhibiting V2-mediated nuclear export of V1 and V1-viral DNA complexes. TYLCV V2 is an important symptom determinant that appears to sequester SlGRXC6 from its association with SlNTRC80, causing dwarf symptoms in plant. Our work, therefore, extended and complemented the current understanding of V2’s role as a symptom determinant and as a target for host defense, among other important roles in geminivirus infection. Given the fact that transient overexpression of SlGRXC6 promoted plant growth, inhibited viral infection, and delayed symptom development, our work also points out a potential antiviral strategy by overexpressing SlGRXC6 in tomato plants.

## Materials and methods

### Plant materials and growth conditions

All agroinfiltration experiments were performed in wild-type (WT) or H2B-RFP (red fluorescent protein fused to the C terminus of histone 2B) [70] transgenic *N*. *benthamiana*. Plants were grown in a growth chamber with temperatures at 26°C (16 h, light) and 22°C (8 h, dark) for 4–6 weeks before being infiltrated with agrobacterium cultures. After infiltration, the plants were kept under the same growth conditions.

### Plasmid construction

The open reading frames (ORFs) of TYLCV V2 (MH205950) [71], SlGRXC6 (XM_004251147), and SlNTRC80 (XM_004249211) were amplified from the cDNA of a TYLCV-infected tomato plant from Jiangsu Province, China, using corresponding primers (S1 Table). Mutants of SlGRXC6^T45A^, SlGRXC6^T53A^, SlGRXC6^G56A^, SlGRXC6^C58A^, SlGRXC6^S69A^, SlGRXC6^S72A^ were synthesized (Invitrogen, China) and confirmed by sequencing.

To investigate subcellular localization, the ORFs of TYLCV V2 (*Bgl*II), SlGRXC6 (*Bam*HI) and SlNTRC80 (*Bam*HI) were amplified using specific primers (S1 Table), individually inserted into the *Bam*HI site of the p1300-YFP vector [29] in frame with and downstream of YFP to generate V2-YFP, SlGRXC6-YFP, and SlNTRC80-YFP.

To make BiFC vectors, SlGRXC6 ORF was cloned into the *Bam*HI site as a fusion with the N-terminal fragment of YFP and V2 ORF was cloned into the *Bam*HI site as a fusion with the C-terminal fragment of YFP, resulting in nYFP-SlGRXC6 and cYFP-V2.

FLAG tagged V2 and SlGRXC6 ORFs were inserted into the *Bam*HI site between the 35S promoter and the 35S terminator in the pCambia1300 binary vector to generate FLAG-V2 and SlGRXC6-FLAG for co-IP experiments.

For the yeast two-hybrid assay, V2 (*Nde*I/*Eco*RI), SlGRXC6 (*Nde*I/*Bam*HI), and SlNTRC80 (*Nde*I/*Bam*HI) ORFs were inserted into the pGADT7 vector or the pGBDT7 vector.

To generate vectors overexpressing SlGRXC6, SlGRXC6 ORF (*Cla*I/*Sal*I) was inserted into the PVX vector. To obtain a construct for virus-induced gene silencing (VIGS) assays, partial coding sequences of SlGRXC6 (encoding amino acids 13 to 113 of SlGRXC6, which is not conserved in the GRX family), or SlNTRC80 (amino acids 150 to 244, which is not conserved in the TRX family) were cloned into the *Eco*RI-*Xho*I sites of a TRV vector [20]. Primers that were used in plasmid construction are shown in S1 Table.

### Agroinfection assays in *N*. *benthamiana* plants

Target vectors were transformed into *A*. *tumefaciens* strain GV3101 by electroporation. Agrobacterial cultures were harvested when the optical density measured at a wavelength of 600 nm (OD_600_) reached approximately 0.8–1.0, then collected by centrifugation, resuspended in induction buffer (10 mM MgSO_4_, 100 mM 2-(N-morpholino) ethanesulfonic acid [pH 5.7], 2 mM acetosyringone), and incubated for more than 2 hours at room temperature. The suspensions were then adjusted to OD_600_ = 0.5 and infiltrated into leaves of 4- to 6-week-old WT or H2B-RFP transgenic *N*. *benthamiana* plants.

### Subcellular localization of proteins

YFP, V2-YFP, SlGRXC6-YFP, SlGRXC6^T53A^-YFP, SlGRXC6^C58A^-YFP, and SlNTRC80-YFP were individually introduced into *A*. *tumefaciens* strain GV3101. Infiltration and subcellular localization observations were performed as described previously [60]. Briefly, we infiltrated *N*. *benthamiana* plants and YFP fluorescence was examined using confocal microscopy (ZEISS LSM 710) at 40 hpai.

### Bimolecular fluorescence complementation (BiFC) assay

nYFP-SlGRXC6 and cYFP-V2 were introduced individually into *A*. *tumefaciens* strain GV3101. BiFC experiments were performed as described previously [60]. Briefly, nYFP-SlGRXC6 and cYFP-V2 were co-infiltrated into *N*. *benthamiana* leaves and YFP fluorescence was observed using confocal microscopy (ZEISS LSM 710) at 48 hpai.

### Co-Immunoprecipitation

The co-IP assay was performed as previously described [29]. Briefly, infiltrated *N*. *benthamiana* leaves were harvested at 40 hpai. Proteins were extracted and incubated with FLAG-conjugated beads (Sigma, USA) and resuspended in 2×SDS loading buffer. Target proteins were detected using a polyclonal anti-GFP antibody (GenScript, USA) or a monoclonal anti-FLAG (Sigma, USA) antibody.

### Yeast two-hybrid screen

RNA was extracted from TYLCV-infected tomato leaves at different developmental stages to construct a yeast two-hybrid library (Invitrogen, China). TYLCV V2 cloned into bait Gal4-BD vector pGBKT7 was transformed into the AH109 yeast strain and used to screen the tomato cDNA library. Mating between bait and prey yeast cells was performed on selective media (SD/–Leu/–Trp/–His) for 3–5 days at 30°C. After the positive clones were cultured, they were transferred to SD/–Leu/–Trp/–His/–Ade with X-α-Gal. The positive clone plasmids were subsequently extracted using the Plasmid Miniprep kit (CoWin Biosciences, China). Following sequencing, a BLAST search was conducted using GenBank (National Center for Biotechnology Information) to determine the associated genes.

The Y2H system was used to examine interactions. SlNTRC80, SlGRXC6 and V2 were cloned into the activation domain (AD)-containing vector or the vector harboring the DNA binding domain (BD). Y2H assays were performed as described previously [60]. Briefly, we transformed constructs into yeast cells and grew them on synthetic defined medium at 30°C for 72 h to test protein-protein interactions.

### Nuclear-cytoplasmic fractionation assay

Nuclear-cytoplasmic fractionation assays were performed as described previously [60]. Briefly, we harvested infiltrated leaves at 40 hpai and separated the nuclear and cytoplasmic fractions. Target proteins were detected using the indicated antibody. Membranes were incubated in Supersignal West Femto substrate (Thermo Scientific) and the protein signals were detected using an Azure C400 ChemiDoc imager. The intensity of signals was quantified by using ImageQuant TL (GE healthcare). Signal areas were automatically identified and the pixel values were measured, and normalized to that of the background signal to obtain the signal intensity. The sum of the protein signal intensity in the cytoplasm and the nucleus was set at 100%. Values represent the average of three plants. Experiments were repeated three times.

PEPC protein and H2B-RFP were used as quality controls for fractionation assays as a cytoplasmic and a nuclear marker, respectively.

### Agrobacterium-mediated TYLCV inoculation

TYLCV infectious clone was constructed as previously reported [29]. The agrobacteria culture was injected into tomato stems with a syringe. The inoculated plants were grown in an insect-free cabinet with supplementary lighting corresponding to a 16-h day length.

### Quantitative PCR analysis

Total RNA was extracted from *SlGRXC6*-scilenced-, *SlNTRC80*-scilenced-, or *SlGRXC6*-overexpressed tomato leaves at different time points using Trizol Reagents (Life Technologies, USA). RNA samples were treated with DNase I and converted to cDNA following manufacturer’s instructions (PrimeScript RT reagent Kit with gDNA Erase, Takara, Japan).

To quantify viral DNA levels by qPCR, total DNA was extracted from mock- or TYLCV-infiltrated tomato leaves at different time points and TYLCV *V1* was detected to represent the viral genomic DNA. The qRCR reaction mixes consisted of 6 μl of SYBR Green supermix (Bio-Rad, USA), 0.10 μl of each primer (10 pmol) and 1.5 μl of DNA or cDNA (10 ng/μl) in a total volume of 12 μl. *SlActin* was used as an internal control for tomato. Each experiment was performed in triplicate and repeated three times. PCR reactions were done in an Applied Biosystems 7500 (ThermoFisher Scietific, USA) real-time PCR detection system. Data analysis was performed using Applied Biosystems 7500 software version 2.0.6.

### Nuclear DNA isolation

Nuclear DNA isolation assays were performed as described previously [72] with minor modifications. Fresh leaves were harvested and mixed with homogenization buffer (1 M hexylene glycol, 10 mM Tris [pH 7.5], 10 mM MgCl_2_, 0.5% triton X-100, and 5 mM β-mercaptoethanol) on ice. The homogenate was transferred to a 50 mL tube and centrifuged at 1,800g for 20 min at 4°C. The pellet was then suspended gently in 10 mL of nuclei wash buffer (0.5 M hexylene glycol, 10 mM Tris [pH 7.5], 10 mM MgCl_2_, 0.5% triton X-100, and 5 mM β-mercaptoethanol) and centrifuged for another 10 minutes at 4°C. The pellet was resuspended with 10 mL of extraction buffer (0.35 M sorbitol, 0.1 M Tris [pH 7.5], 5 mM EDTA, 2% CTAB, 4 M NaCl, 0.4% SDS and 0.1% β-mercaptoethanol) with 10 μg RNase H to remove RNA. The mixture was incubated at 65°C for 30 min and then extracted with chloroform-isoamytalcohol (24:1). After 10 min of centrifugation, the upper phase was mixed with isopropanol and incubate at -20°C for 30 min to precipitate DNA.

### *In vitro* pull-down competition assay

TYLCV V2, SlGRXC6, and SlNTRC80 ORFs were cloned into pET32a, pGEX4T-1, or pHMTC to make constructs expressing His6-, GST-, or MBP-tagged proteins. All constructs were transformed into *E*. *coli* BL21 (DE3) cells and cultured at 37°C. After the OD_600_ had reached ∼0.6, β-D-thiogalactoside (IPTG) was added to the cultures and incubated for 4 hours. Bacterial cells were pelleted, resuspended with phosphate-buffered saline (PBS) buffer, and sonicated to break cells. The His6-, GST-, and MBP-fused proteins were individually purified using a nickel-nitrilotriacetic acid (Ni-NTA) resin binding column (Qiagen, Germany), GST binding column (Qiagen, Germany), and MBP binding column (Novagen, Germany), respectively, according to manufacturer’s instructions. Competitive pulldown assays were performed as described previously [20] with minor modifications. Briefly, the indicated amounts of His6-V2 or His6 were mixed with 2 μg of GST-SlGRXC6 for 1 h before being incubated with 2 μg of MBP-SlNTRC80 for pulldown assays. The column-bound proteins were eluted and detected by immunoblotting with anti-His6 (Abcam, UK), anti-MBP (Sigma, USA), or anti-GST (GenScript, USA) antibody.

## Supporting information

S1 Fig Analysis of transgenic tomato plants expressing the V2 protein of TYLCV. (a) The symptom-like phenotype in V2 transgenic tomato plants. 35S:V2 transgenic tomato plants were generated via *Agrobacterium* transformation. Bar: 5 cm. (b) Detection of V2 transcripts in transgenic tomato plants. Relative *V2* expression levels were determined by qRT-PCR using V2 gene-specific primers, *SlActin* was used as an internal control. Mock represents transgenic tomato plants with an empty vector. (c) Aboveground plant heights of 35S or 35S:V2 transgenic tomato plants. Mock represents transgenic plants that were transformed with an empty vector.
(TIF)

S2 Fig SlGRXC6 is the only member of the tomato GRX family that interacts with V2 with high affinity. (a) Phylogeny analysis of the GRX gene family in tomato and *Arabidopsis thaliana*. The phylogenetic tree was constructed using MEGA 5.05 (Neighbor–Joining method). (b) Y2H assay of the interaction between V2 and tomato GRXs. Yeast cells co-transformed with the indicated plasmids were spotted on media without (SD/-Trp) or with selection (SD/-His/-Leu/-Trp) to screen for positive interactions.
(TIF)

S3 Fig Silencing of PDS expression in tomato plants. (a) Phenotype of tomato plants in which the *SlPDS* gene was silenced (TRV-SlPDS). (b) Relative expression levels of *SlPDS* in VIGS-treated and control tomato (TRV) as determined by qRT-PCR. *SlActin* was used as an internal control. Each dataset was derived from at least three biological repeats. The transcript levels of *SlPDS* were tested at 12 dpai.
(TIF)

S4 Fig The effect of knocking down tomato GRXs on TYLCV infections. (a) The growth phenotypes of tomato plants in which gene expression of *SlGRX25*, *SlGRX38*, *SlGRX39* or *SlGRXC6* was silenced (TRV-SlGRX25, TRV-SlGRX38, TRV-SlGRX39 or TRV-SlGRXC6) at 16 dpai. Bar: 5 cm. (b) The relative levels of *SlGRX25*, *SlGRX38*, *SlGRX39* or *SlGRXC6* transcripts in control (TRV) and knockdown (TRV-SlGRXC6, SlGRX25, SlGRX38 or SlGRX39) tomato plants were determined by qRT-PCR at 12 dpai. *SlActin* was used as an internal control. Student’s t test was performed, and asterisks indicate a significant difference (P < 0.05). Each data set was derived from three independent plants. (c) The aboveground heights of TRV-SlGRX25, TRV-SlGRX38, TRV-SlGRX39, TRV-SlGRXC6 or TRV plants were measured at 16 dpai. (d) Symptoms caused by TYLCV infection in *SlGRX25*-, *SlGRX38*-, *SlGRX39*-, *SlGRXC6*-silenced or control plants. Leaves were photographed at 23 dpi. Bar: 5 cm. (e) The accumulated viral genomic DNA in systemic leaves as measured by qPCR. Accumulated levels of viral genomic DNA were tested in *SlGRX25*-, *SlGRX38*-, *SlGRX39*-, *SlGRXC6*-silenced or control tomato plants infected with TYLCV at 23 dpi as shown in Fig 2A. Experiments were repeated twice with similar results.
(TIF)

S5 Fig The subcellular distribution of V2 in the presence of SlGRXC6. Both V2-RFP and SlGRXC6-YFP were expressed and detected in *N*. *benthamiana* cells. Bars: 50 μm. Experiments were repeated three times.
(TIF)

S6 Fig Confirmation of DNA quality isolated from plant nuclei. Total and nuclear DNA was extracted from tomato plants with SlGRXC6 overexpressed or silenced. PCR amplification was conducted by using gene-specific primers of PCNA, a nuclear gene, and COX1, a mitochondrial gene. N: genomic DNA extracted from the nucleus; T: total genomic DNA extracted from tomato leaves.
(TIF)

S7 Fig SlGRXC6 restricts TYLCV viral genomic DNA nucleocytoplasmic shuttling. (a) Percentage of the accumulated viral genomic DNA in the nucleus fraction in tomato plants in the absence or presence of overexpressed SlGRXC6 at 23 dpi. Viral accumulation was assessed in three plants by qPCR. Data are means ± SD. Asterisks on the top of the bars indicate significant differences (student’s t-test). Experiments were repeated twice with similar results. (b) Percentages of the accumulated viral genomic DNA in the nucleus fraction in tomato plants without or with *SlGRXC6* knocked down as in a.
(TIF)

S8 Fig Expression of plant defense-related genes in TYLCV infected, SlGRXC6, SlGRX38 or SlGRX39-silenced plants. The relative levels of *PR1-a*, *GLUA*, *CHI3* transcripts were tested in TYLCV-inoculated *SlGRXC6*, *SlGRX38*, *SlGRX39*-silenced or control tomato plants as shown in Fig 2A. Data are means ± SD (n = 3). Experiments were repeated twice with similar results.
(TIF)

S9 Fig Identification of the interaction between SlGRXC6 and SlTRX1-140/SlNTRC80. (a) A diagram illustrating the tomato TRX proteins that are predicted to be associated with SlGRXC6 based on prediction by the STRING program. Arrow points to SlGRXC6. (b) The interactions of SlGRXC6-SlTRX1-140 and SlGRXC6-SlNTRC80 were confirmed using Y2H. Yeast cells co-transformed with the indicated constructs were subjected to 10-fold serial dilutions and grown on selection medium (SD/-His/-Leu/-Trp).
(TIF)

S10 Fig Localization of YFP and SlNTRC80-YFP in H2B-RFP transgenic N. benthamiana plants. The H2B-RFP signal represents the nucleus. Bars: 50 μm.
(TIF)

S11 Fig Expression of SlNTRC80 is affected by SlGRXC6. (a) The relative levels of *SlNTRC80* transcripts were tested using qRT-PCR. Total RNA was extracted from newly emerged systemic leaves in PVX- or PVX-SlGRXC6-treated plants. The expression of *SlNTRC80* was tested as shown in Fig 2A. Data are means ± SD (n = 3). (b) Relative levels of *SlNTRC80* transcripts were tested using qRT-PCR. Total RNA was extracted from newly emerged systemic leaves from TRV- and TRV-SlGRXC6-treated plants. The expression of *SlNTRC80* was tested as shown in Fig 2A. Data are means ± SD (n = 3).
(TIF)

S12 Fig Interactions between SlNTRC80 and SlGRXC6T53A or SlGRXC6C58A. The co-IP assay was used to test whether SlNTRC80 interacted with SlGRXC6^T53A^ or SlGRXC6^C58A^. The assay was performed as shown in Fig 1C.
(TIF)

S13 Fig TYLCV infection promotes the expression of SlGRXC6. (a) The expression of SlGRXC6 in healthy or TYLCV-infected *N*.*benthamiana* cells. The expressed SlGRXC6-YFP in epidermal cells of *N*.*benthamiana* leaves was detected either by confocal microscopy (left panel) or by western blotting using an anti-GFP polyclonal antibody (right panel). Experiments were repeated three times. (b) The relative levels of *SlGRXC6* transcripts in plants as measured by qRT-PCR. Accumulated levels of *SlGRXC6* transcript were tested in TYLCV- or mock-inoculated plants at 23 dpi as Fig 2A. Data are means ± SD (n = 3). Experiments were repeated three times with similar results.
(TIF)

S1 Table Primers used in this study. (DOCX)
