# Supplementary figures and images for: Host GRXC6 restricts Tomato yellow leaf curl virus infection by inhibiting the nuclear export of the V2 protein
Source: PLoS Pathog. 2021 Aug 16;17(8):e1009844. doi: 10.1371/journal.ppat.1009844 (PMC8389846; doi:10.1371/journal.ppat.1009844)

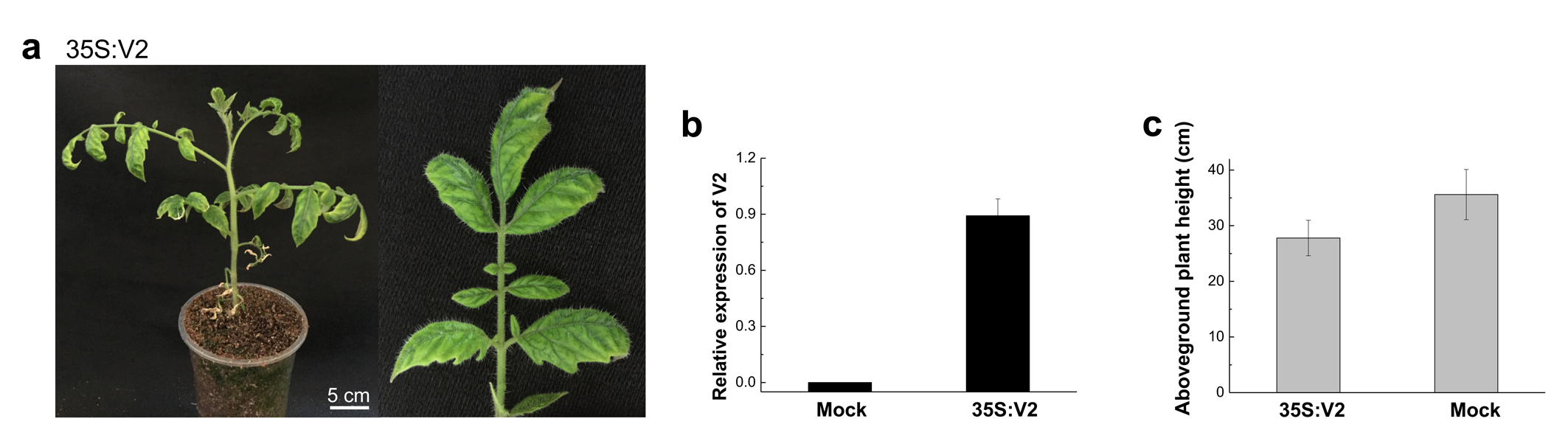

Supplement: S1 Fig — (a) The symptom-like phenotype in V2 transgenic tomato plants. 35S:V2 transgenic tomato plants were generated via Agrobacterium transformation. Bar: 5 cm. (b) Detection of V2 transcripts in transgenic tomato plants. Relative V2 expression levels were determined by qRT-PCR using V2 gene-specific primers, SlActin was used as an internal control. Mock represents transgenic tomato plants with an empty vector. (c) Aboveground plant heights of 35S or 35S:V2 transgenic tomato plants. Mock represents transgenic plants that were transformed with an empty vector. (TIF) [file ppat.1009844.s001.tif]

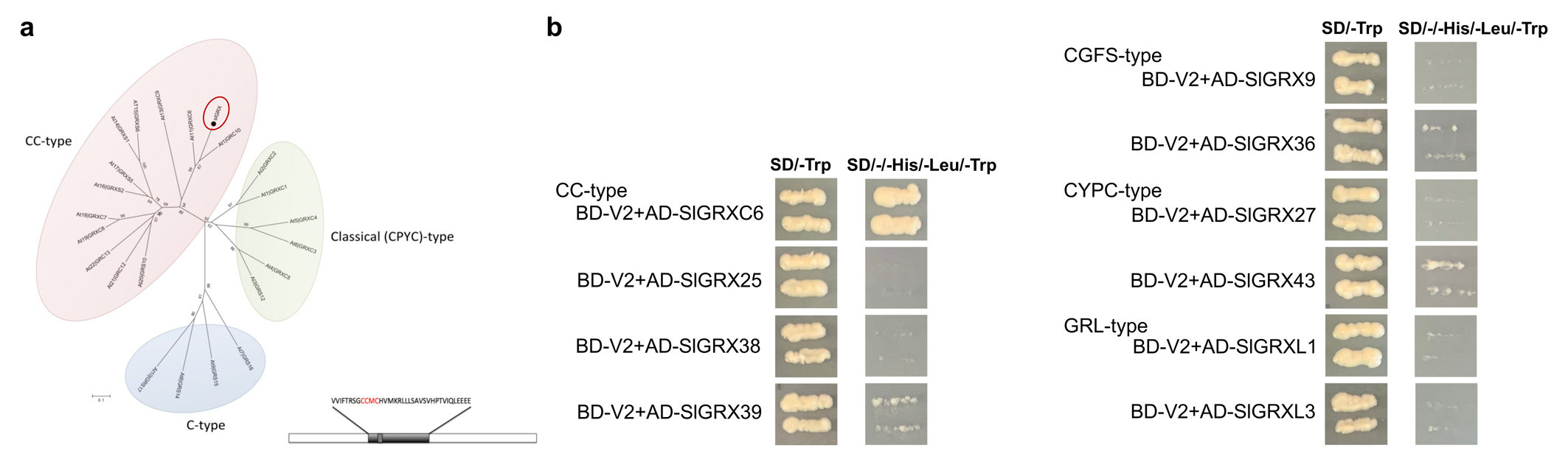

Supplement: S2 Fig — (a) Phylogeny analysis of the GRX gene family in tomato and Arabidopsis thaliana. The phylogenetic tree was constructed using MEGA 5.05 (Neighbor–Joining method). (b) Y2H assay of the interaction between V2 and tomato GRXs. Yeast cells co-transformed with the indicated plasmids were spotted on media without (SD/-Trp) or with selection (SD/-His/-Leu/-Trp) to screen for positive interactions. (TIF) [file ppat.1009844.s002.tif]

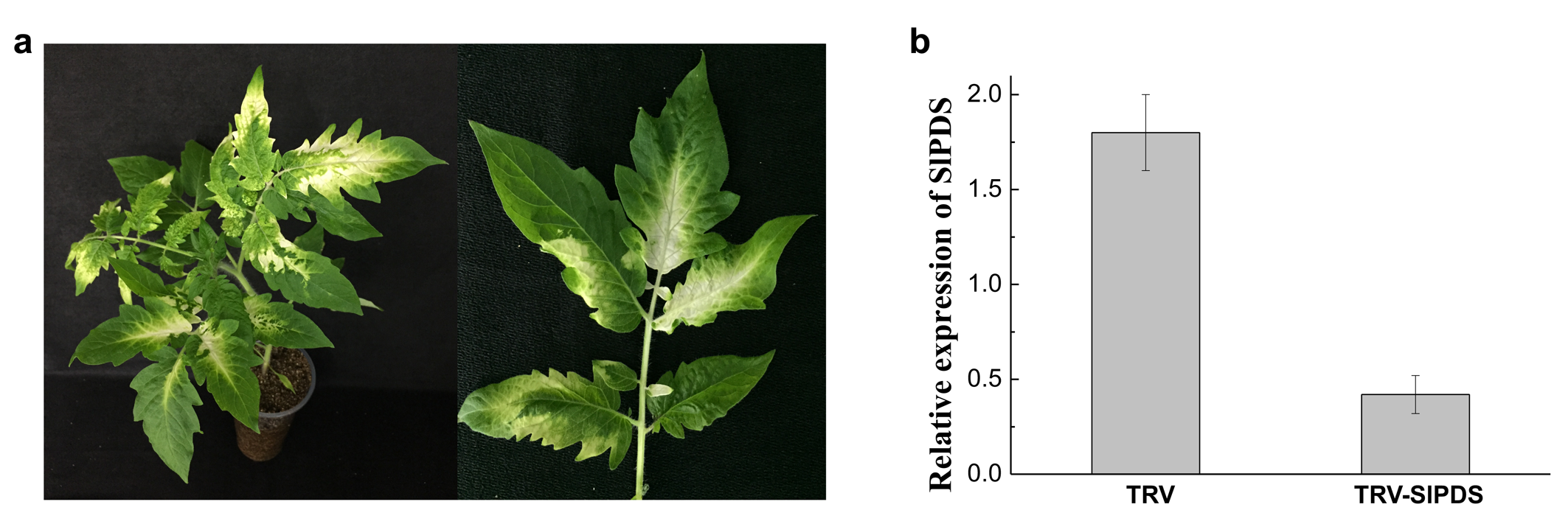

Supplement: S3 Fig — (a) Phenotype of tomato plants in which the SlPDS gene was silenced (TRV-SlPDS). (b) Relative expression levels of SlPDS in VIGS-treated and control tomato (TRV) as determined by qRT-PCR. SlActin was used as an internal control. Each dataset was derived from at least three biological repeats. The transcript levels of SlPDS were tested at 12 dpai. (TIF) [file ppat.1009844.s003.tif]

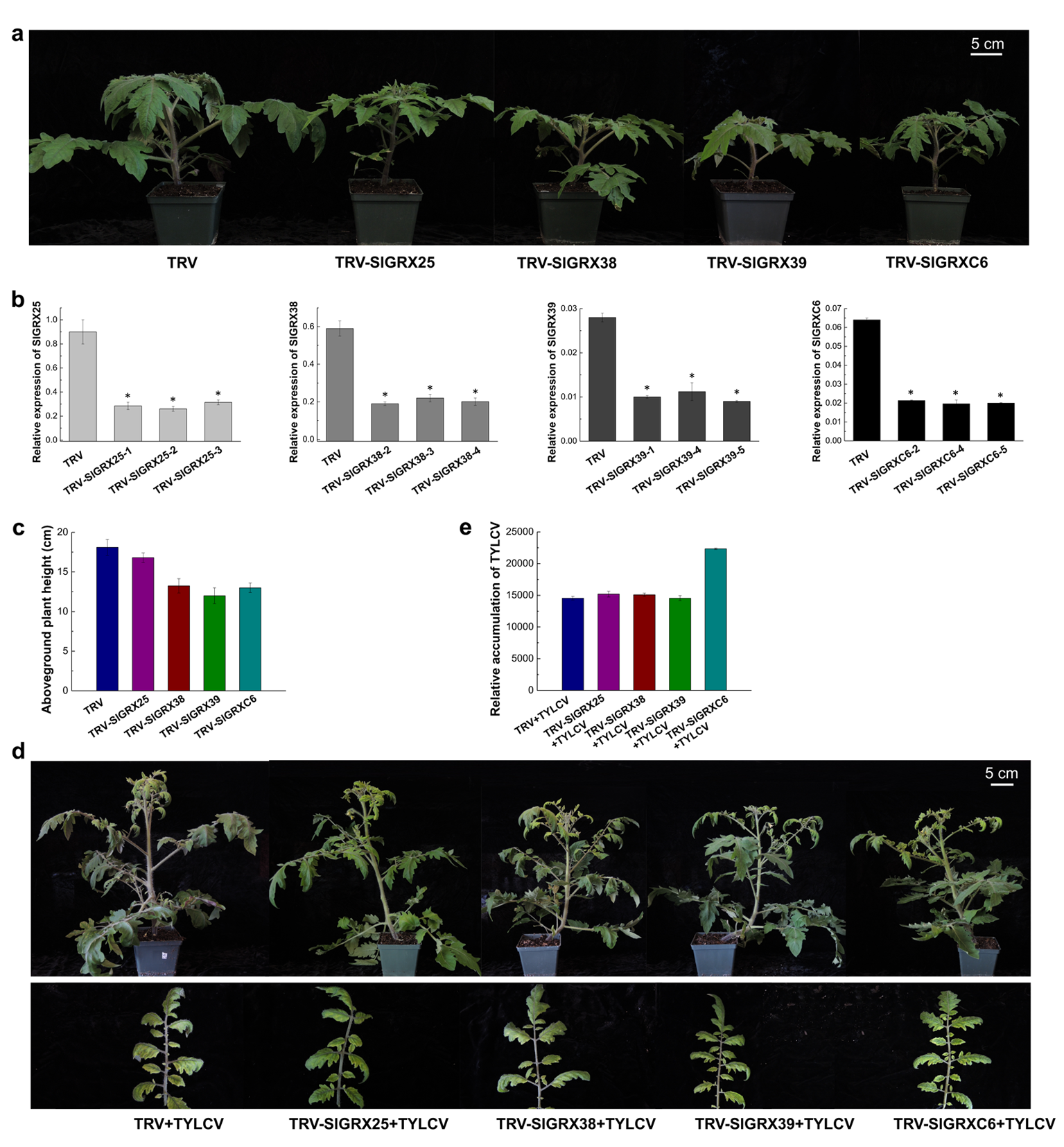

Supplement: S4 Fig — (a) The growth phenotypes of tomato plants in which gene expression of SlGRX25, SlGRX38, SlGRX39 or SlGRXC6 was silenced (TRV-SlGRX25, TRV-SlGRX38, TRV-SlGRX39 or TRV-SlGRXC6) at 16 dpai. Bar: 5 cm. (b) The relative levels of SlGRX25, SlGRX38, SlGRX39 or SlGRXC6 transcripts in control (TRV) and knockdown (TRV-SlGRXC6, SlGRX25, SlGRX38 or SlGRX39) tomato plants were determined by qRT-PCR at 12 dpai. SlActin was used as an internal control. Student’s t test was performed, and asterisks indicate a significant difference (P < 0.05). Each data set was derived from three independent plants. (c) The aboveground heights of TRV-SlGRX25, TRV-SlGRX38, TRV-SlGRX39, TRV-SlGRXC6 or TRV plants were measured at 16 dpai. (d) Symptoms caused by TYLCV infection in SlGRX25-, SlGRX38-, SlGRX39-, SlGRXC6-silenced or control plants. Leaves were photographed at 23 dpi. Bar: 5 cm. (e) The accumulated viral genomic DNA in systemic leaves as measured by qPCR. Accumulated levels of viral genomic DNA were tested in SlGRX25-, SlGRX38-, SlGRX39-, SlGRXC6-silenced or control tomato plants infected with TYLCV at 23 dpi as shown in Fig 2A. Experiments were repeated twice with similar results. (TIF) [file ppat.1009844.s004.tif]

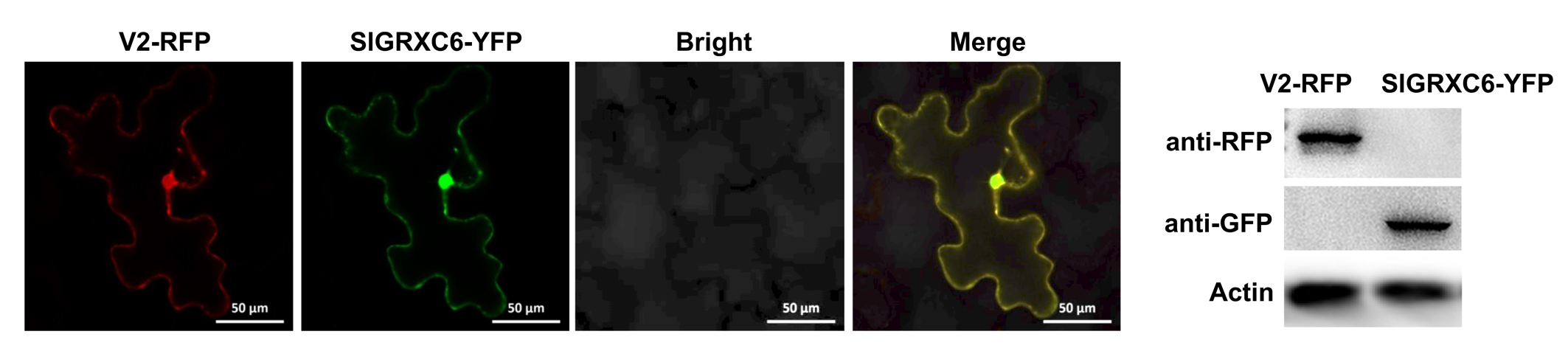

Supplement: S5 Fig — Both V2-RFP and SlGRXC6-YFP were expressed and detected in N. benthamiana cells. Bars: 50 μm. Experiments were repeated three times. (TIF) [file ppat.1009844.s005.tif]

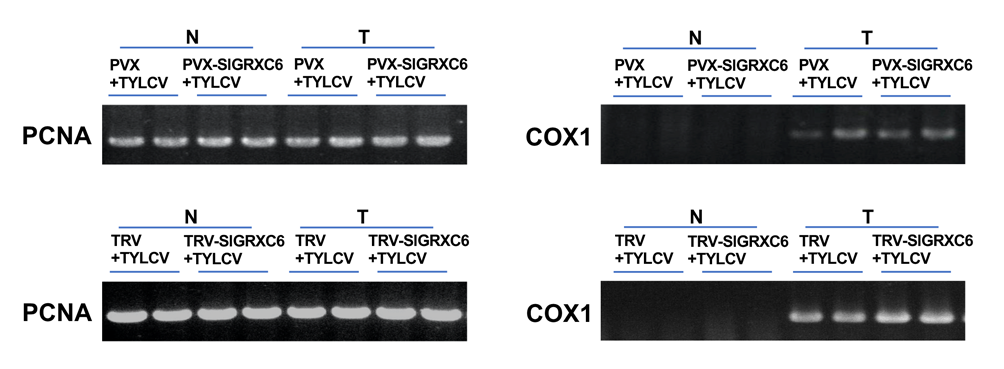

Supplement: S6 Fig — Total and nuclear DNA was extracted from tomato plants with SlGRXC6 overexpressed or silenced. PCR amplification was conducted by using gene-specific primers of PCNA, a nuclear gene, and COX1, a mitochondrial gene. N: genomic DNA extracted from the nucleus; T: total genomic DNA extracted from tomato leaves. (TIF) [file ppat.1009844.s006.tif]

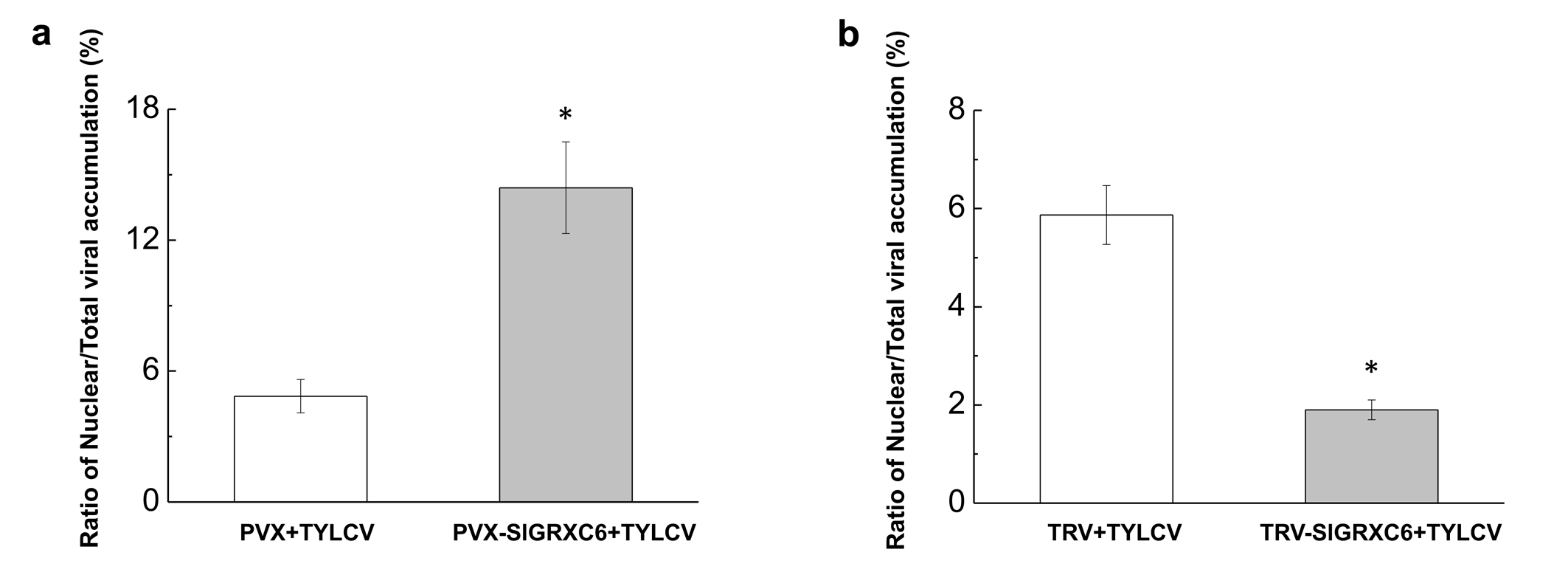

Supplement: S7 Fig — (a) Percentage of the accumulated viral genomic DNA in the nucleus fraction in tomato plants in the absence or presence of overexpressed SlGRXC6 at 23 dpi. Viral accumulation was assessed in three plants by qPCR. Data are means ± SD. Asterisks on the top of the bars indicate significant differences (student’s t-test). Experiments were repeated twice with similar results. (b) Percentages of the accumulated viral genomic DNA in the nucleus fraction in tomato plants without or with SlGRXC6 knocked down as in a. (TIF) [file ppat.1009844.s007.tif]

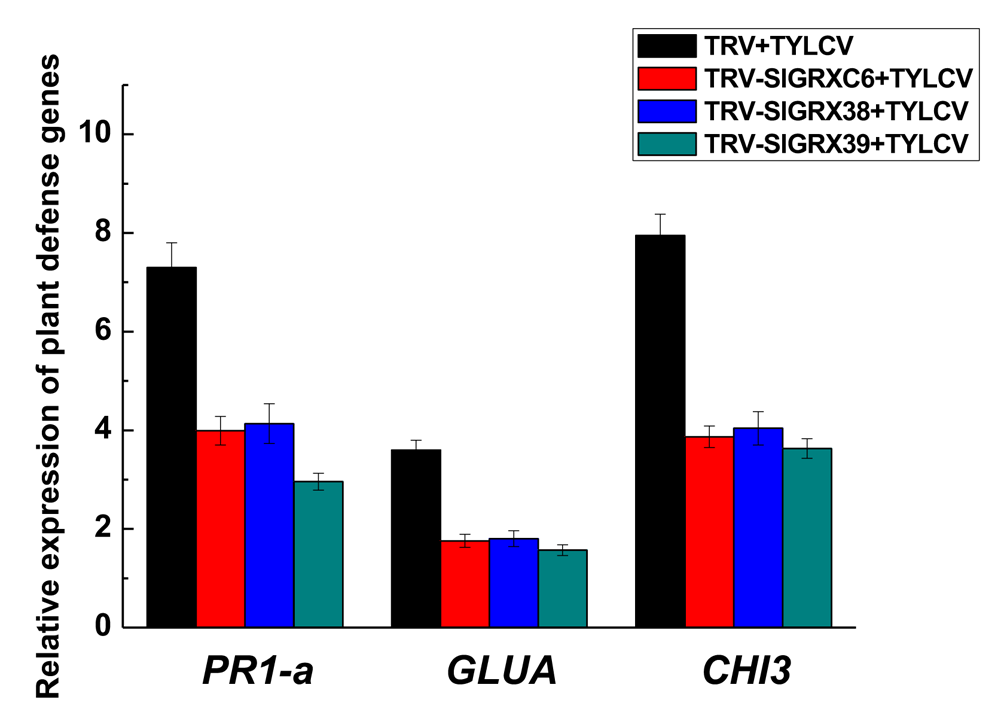

Supplement: S8 Fig — The relative levels of PR1-a, GLUA, CHI3 transcripts were tested in TYLCV-inoculated SlGRXC6, SlGRX38, SlGRX39-silenced or control tomato plants as shown in Fig 2A. Data are means ± SD (n = 3). Experiments were repeated twice with similar results. (TIF) [file ppat.1009844.s008.tif]

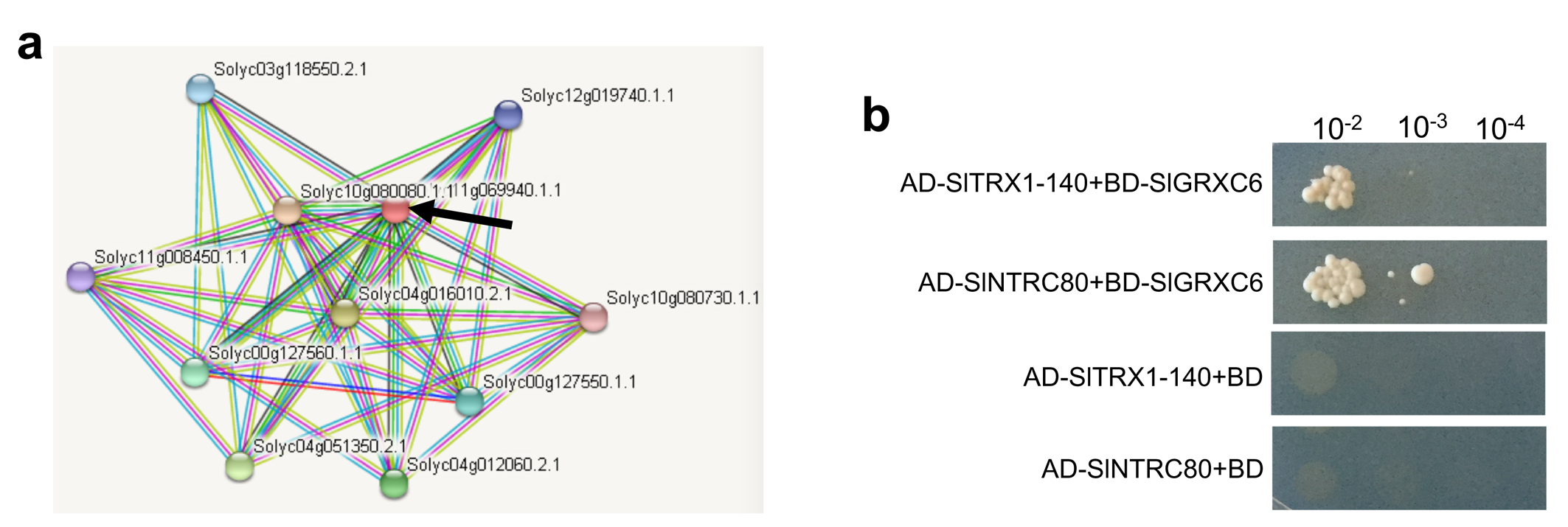

Supplement: S9 Fig — (a) A diagram illustrating the tomato TRX proteins that are predicted to be associated with SlGRXC6 based on prediction by the STRING program. Arrow points to SlGRXC6. (b) The interactions of SlGRXC6-SlTRX1-140 and SlGRXC6-SlNTRC80 were confirmed using Y2H. Yeast cells co-transformed with the indicated constructs were subjected to 10-fold serial dilutions and grown on selection medium (SD/-His/-Leu/-Trp). (TIF) [file ppat.1009844.s009.tif]

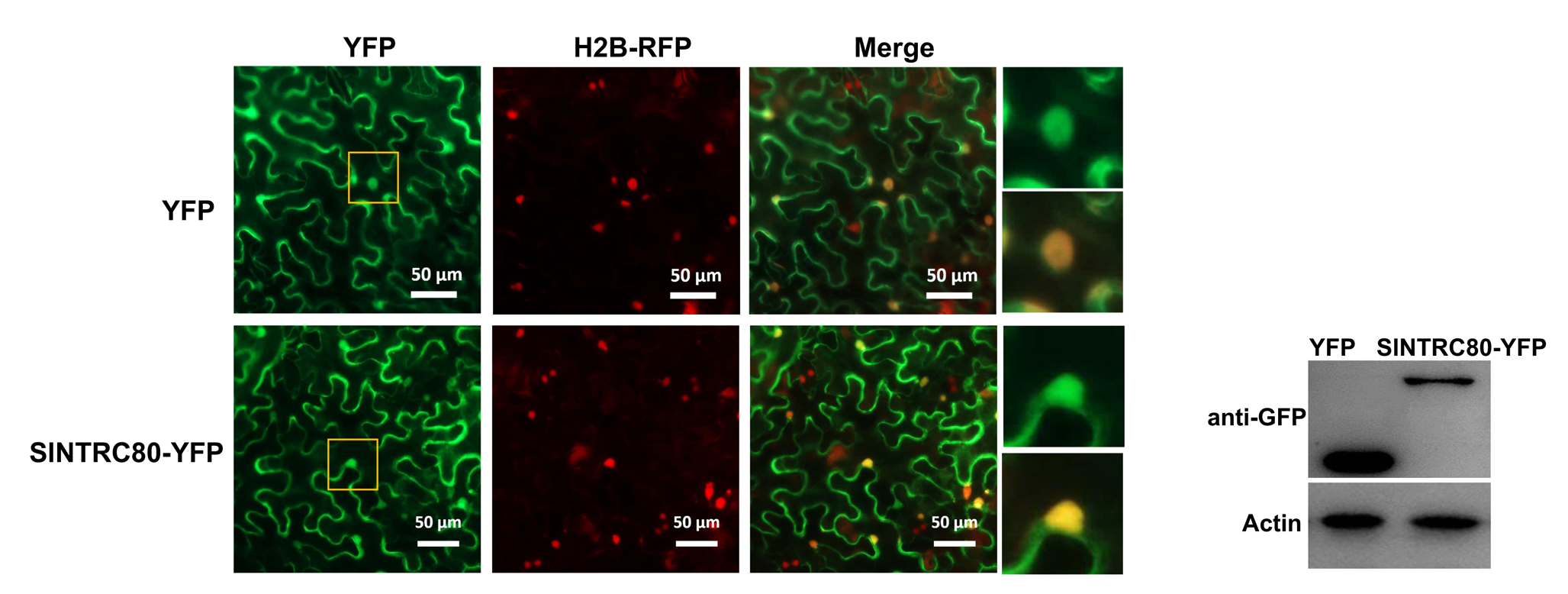

Supplement: S10 Fig — The H2B-RFP signal represents the nucleus. Bars: 50 μm. (TIF) [file ppat.1009844.s010.tif]

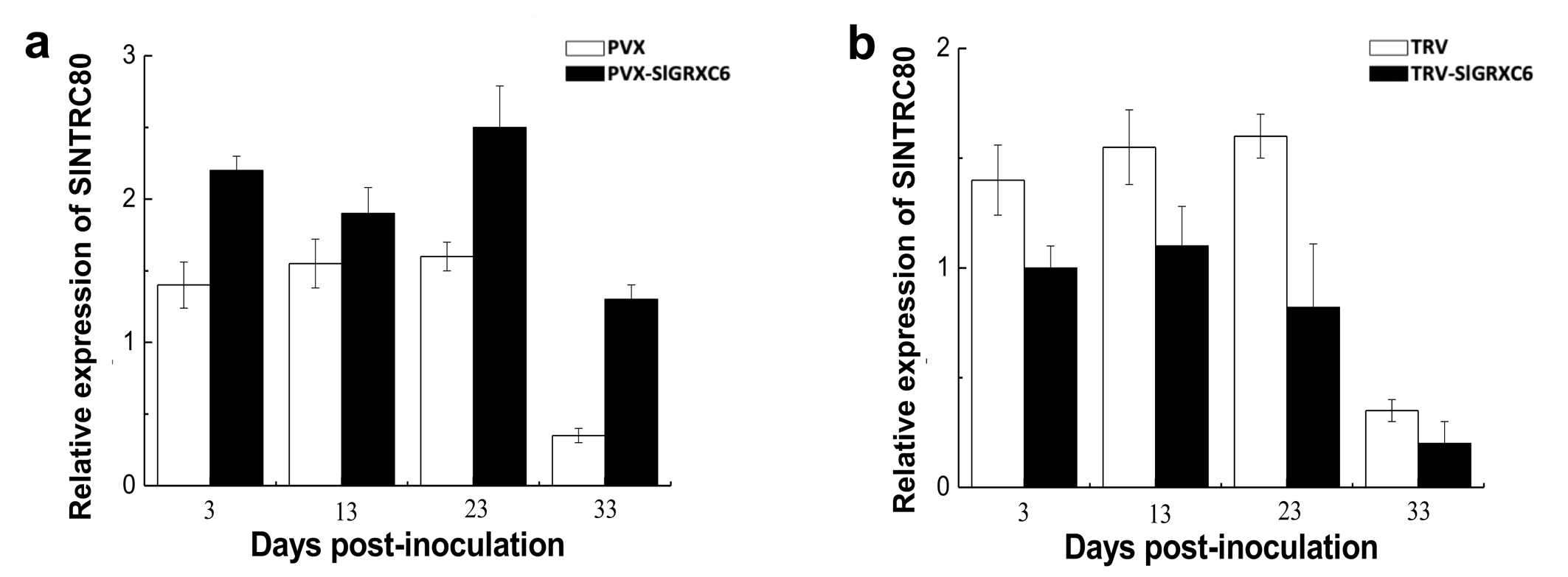

Supplement: S11 Fig — (a) The relative levels of SlNTRC80 transcripts were tested using qRT-PCR. Total RNA was extracted from newly emerged systemic leaves in PVX- or PVX-SlGRXC6-treated plants. The expression of SlNTRC80 was tested as shown in Fig 2A. Data are means ± SD (n = 3). (b) Relative levels of SlNTRC80 transcripts were tested using qRT-PCR. Total RNA was extracted from newly emerged systemic leaves from TRV- and TRV-SlGRXC6-treated plants. The expression of SlNTRC80 was tested as shown in Fig 2A. Data are means ± SD (n = 3). (TIF) [file ppat.1009844.s011.tif]

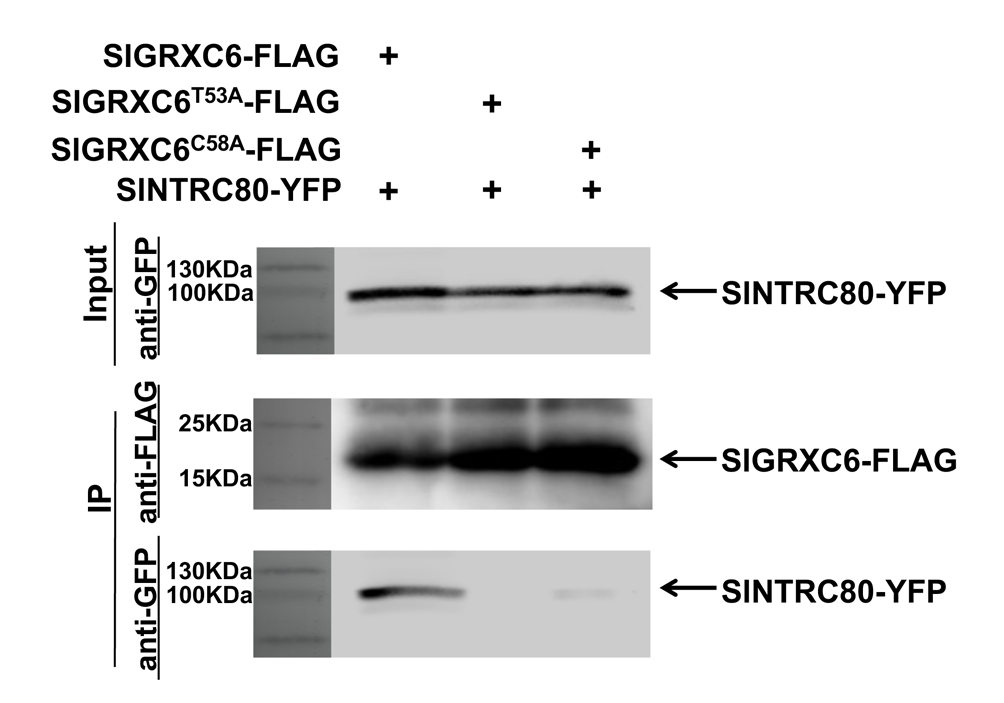

Supplement: S12 Fig — The co-IP assay was used to test whether SlNTRC80 interacted with SlGRXC6T53A or SlGRXC6C58A. The assay was performed as shown in Fig 1C. (TIF) [file ppat.1009844.s012.tif]

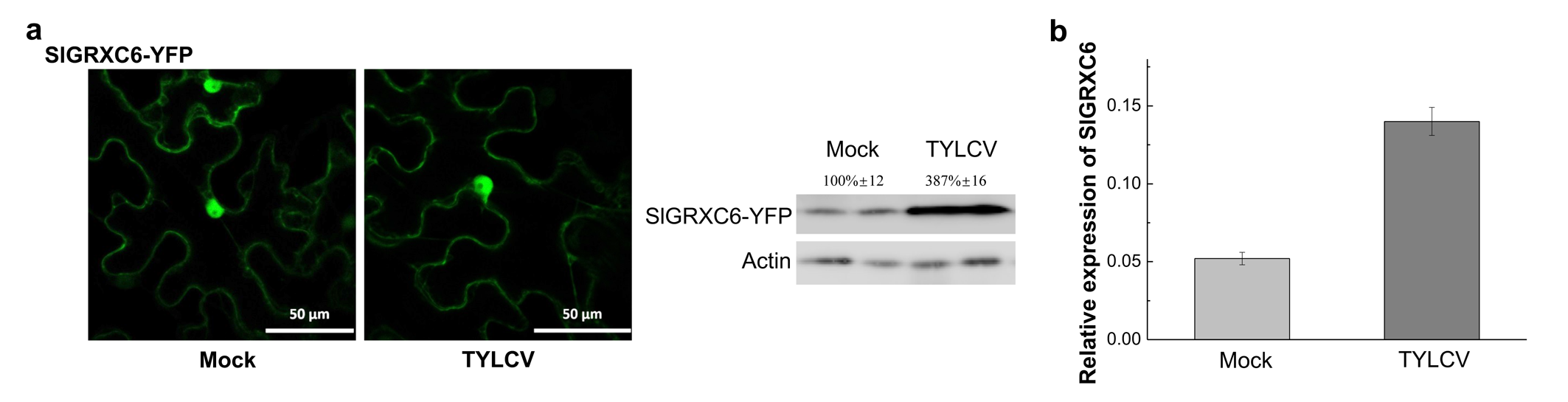

Supplement: S13 Fig — (a) The expression of SlGRXC6 in healthy or TYLCV-infected N.benthamiana cells. The expressed SlGRXC6-YFP in epidermal cells of N.benthamiana leaves was detected either by confocal microscopy (left panel) or by western blotting using an anti-GFP polyclonal antibody (right panel). Experiments were repeated three times. (b) The relative levels of SlGRXC6 transcripts in plants as measured by qRT-PCR. Accumulated levels of SlGRXC6 transcript were tested in TYLCV- or mock-inoculated plants at 23 dpi as Fig 2A. Data are means ± SD (n = 3). Experiments were repeated three times with similar results. (TIF) [file ppat.1009844.s013.tif]
