# Supplementary material for: Host GRXC6 restricts Tomato yellow leaf curl virus infection by inhibiting the nuclear export of the V2 protein
Source: PLoS Pathog. 2021 Aug 16;17(8):e1009844. doi: 10.1371/journal.ppat.1009844 (PMC8389846; doi:10.1371/journal.ppat.1009844)
Supplement: S1 Table — (DOCX) [file ppat.1009844.s014.docx]

**Supporting Information**

**Supplementary Table**

S1 Table Primers used in this study

|  | Designation | Sequence(5’ to 3’) | Assay |
| --- | --- | --- | --- |
| 1 | TY-V2-F-AB | CGCCATATGATGTGGGATCCACTTCTAAAT | BD-V2 |
| 2 | TY-V2-R-AB | CGGAATTCTCAGGGCTTCGATACATTCT | BD-V2 |
| 3 | GRX-NF | CGCCATATGCAAGGCGTTCGCCGTTAC | AD-SlGRXC6,  BD-SlGRXC6,  GST-SlGRXC6 |
| 4 | GRX-BR | CGGGATCCTTACAATACCATTTCAGTAATCGCACC | AD-SlGRXC6,  BD-SlGRXC6  GST-SlGRXC6 |
| 5 | NTRC80-NF | CGCCATATGATGGCTAATACTACTCCCAATATT | AD-SlNTRC80,  MBP-SlNTRC80 |
| 6 | NTRC80-BR | CGGGATCCTCACTTATTTGCTTCAATAAACTC | AD-SlNTRC80,  MBP-SlNTRC80 |
| 7 | V2-bgF | GAAGATCTATGTGGGATCCACTTCTAAAT | V2-YFP,  cYFP-V2, |
| 8 | V2-bgR | GAAGATCTGGGCTTCGATACATTCTGTAT | V2-YFP,  cYFP-V2,  FLAG-V2 |
| 9 | FLAG-V2-F | GAAGATCTATGGATTACAAGGATGATGATGATAATGTGGGATCCACTTCTAAATG | FLAG-V2 |
| 10 | GRX-bF | CGGGATCCATGCAAGGCGTTCGCCGTTAC | nYFP-SlGRXC6,  SlGRXC6-YFP,  SlGRXC6T53A-YFP,  SlGRXC6C58A-YFP,  SlGRXC6-FLAG,  SlGRXC6T53A-FLAG,  SlGRXC6C58A-FLAG |
| 11 | GRX-bR | CGGGATCCCAATACCATTTCAGTAATCGCACC | nYFP-SlGRXC6,  SlGRXC6-YFP,  SlGRXC6T53A-YFP,  SlGRXC6C58A-YFP |
|  | FLAG-GRX-R | CGGGATCCTATCATCATCATCCTTGTAATCCATCAATACCATTTCAGTAATCGCACC | SlGRXC6-FLAG,  SlGRXC6T53A-FLAG,  SlGRXC6C58A-FLAG |
| 12 | NTRC80-bF | CGGGATCCATGGCTAATACTACTCCCAATATT | SlNTRC80-YFP |
| 13 | NTRC80-bR | CGGGATCCCTTATTTGCTTCAATAAACTC | SlNTRC80-YFP |
| 14 | GRX-cF | CCATCGATATGCAAGGCGTTCGCCGTTAC | PVX-SlGRXC6 |
| 15 | GRX-sR | GCGTCGACCAATACCATTTCAGTAATCGCACC | PVX-SlGRXC6 |
| 16 | GRX-eF | CGGAATTCATGCAAGGCGTTCGCCGTTAC | TRV-SlGRXC6 |
| 17 | GRX-xhR | CCCTCGAGCAATACCATTTCAGTAATCGCACC | TRV-SlGRXC6 |
| 18 | NTRC80-TF | ATTTCCTGAGGGATAACTGGA | TRV-SlNTRC80 |
| 19 | NTRC80-TR | GACCCTTGAAAAGTGGTGAT | TRV-SlNTRC80 |
